# A survey of fecal virome and bacterial community of the diarrhea-affected cattle in northeast China reveals novel disease-associated ecological risk factors

**DOI:** 10.1128/msystems.00842-23

**Published:** 2023-12-18

**Authors:** Qinghe Zhu, Shanshan Qi, Donghua Guo, Chunqiu Li, Mingjun Su, Jianfa Wang, Zijian Li, Dan Yang, Haibo Sun, Xiaoran Wang, Meijiao Wang, Haoyang Wu, Shiping Yu, Wenfei Bai, Yongchen Zhang, Xu Yang, Limin Jiang, Jiaying Liu, Yingying Zhao, Xiaoxu Xing, Da Shi, Li Feng, Dongbo Sun

**Affiliations:** 1 Key Laboratory of Bovine Disease Control in Northeast China, Ministry of Agriculture and Rural affairs of the People’s Republic of China, Heilongjiang Provincial Key Laboratory of Prevention and Control of Bovine Diseases, College of Animal Science and Veterinary Medicine, Heilongjiang Bayi Agricultural University, Daqing, China; 2 State Key Laboratory of Veterinary Biotechnology, Harbin Veterinary Research Institute, Chinese Academy of Agricultural Sciences, Harbin, China; University of Illinois at Chicago, Chicago, Illinois, USA

**Keywords:** cattle, diarrhea, disease ecology, virome, bacterial community

## Abstract

Limited information on the virome and bacterial community hampers our ability to discern systemic ecological risk factors that cause cattle diarrhea, which has become a pressing issue in the control of disease. A total of 110 viruses, 1,011 bacterial genera, and 322 complete viral genomes were identified from 70 sequencing samples mixed with 1,120 fecal samples from 58 farms in northeast China. For the diarrheic samples, the identified virome and bacterial community varied in terms of composition, abundance, diversity, and geographic distribution in relation to different disease-associated ecological factors; the abundance of identified viruses and bacteria was significantly correlated with the host factors of clinical status, cattle type, and age, and with environmental factors such as aquaculture model and geographical location (*P* < 0.05); a significant interaction occurred between viruses and viruses, bacteria and bacteria, as well as between bacteria and viruses (*P* < 0.05). The abundance of *SMB53*, *Butyrivibrio*, *Facklamia*, *Trichococcus*, and *Turicibacter* was significantly correlated with the health status of cattle (*P* < 0.05). The proportion of BRV, BCoV, BKV, BToV, BoNoV, BoNeV, BoAstV, BEV, BoPV, and BVDV in 1,120 fecal samples varied from 1.61% to 12.05%. A series of significant correlations were observed between the prevalence of individual viruses and the disease-associated ecological factors. A genome-based phylogenetic analysis revealed high variability of 10 bovine enteric viruses. The bovine hungarovirus was initially identified in both dairy and beef cattle in China. This study elucidates the fecal virome and bacterial community signatures of cattle affected by diarrhea, and reveals novel disease-associated ecological risk factors, including cattle type, cattle age, aquaculture model, and geographical location.

**IMPORTANCE:**

The lack of data on the virome and bacterial community restricts our capability to recognize ecological risk factors for bovine diarrhea disease, thereby hindering our overall comprehension of the disease’s cause. In this study, we found that, for the diarrheal samples, the identified virome and bacterial community varied in terms of composition, abundance, diversity, configuration, and geographic distribution in relation to different disease-associated ecological factors. A series of significant correlations were observed between the prevalence of individual viruses and the disease-associated ecological factors. Our study aims to uncover novel ecological risk factors of bovine diarrheal disease by examining the pathogenic microorganism-host-environment disease ecology, thereby providing a new perspective on the control of bovine diarrheal diseases.

## INTRODUCTION

Cattle, as a highly varied and widespread species, are the most commonly raised ruminants on a global scale. They possess a significant connection to human existence, providing us with essential livestock products such as milk, meat, and fur, as well as serving as a source of farming power (1). The intricate ecology of diseases, including pathogenic microorganisms, geographical environment, and climate, has caused the frequent occurrence of diseases in cattle breeding, thus impeding the healthy development of the cattle industry. The diarrheic disease is the most common and severe cattle disorder, with an incidence rate ranging from 29% to 58% (2, 3). This disease is the primary source of calf death, causing considerable financial losses to the cattle industry (4). Simultaneously, several pathogenic microorganisms responsible for bovine diarrheic disease have the potential to spread to other species and infect humans, posing a significant risk to human health, food security, and public well-being. These include, but are not limited to, *Escherichia coli*, rotavirus, coronavirus, *Cryptosporidium parvum*, and others (5–7). Cattle suffering from diarrhea experience a decrease in production and reproductive performance, as well as economic benefits. This leads to an increase in methane emissions due to the expansion of the breeding population, an increase in antibiotic residues due to the use of antibiotics, and a loss of resources due to reduced feed utilization. This excessive consumption will have adverse effects on the environment.

Bovine diarrheic disease is mainly caused by the interplay between pathogen, host, and environment, of which pathogenic microorganism infection is the primary factor. In recent years, numerous studies have been conducted to investigate the microorganisms that cause bovine diarrhea. A variety of pathogenic microorganisms have been identified, such as the bovine rotavirus (BRV), bovine coronavirus (BCoV), bovine norovirus (BoNoV), bovine enterovirus (BEV), bovine viral diarrhea virus (BVDV), *E. coli*, *Clostridium perfringens*, *Salmonella*, and more (8, 9). These findings have served as a basis for understanding, preventing, and controlling bovine diarrheic disease. Despite this, the interaction and synergistic pathogenicity between microorganisms, the association between pathogenic microorganism infection and host factors including clinical status, breed, and age, as well as the correlation between pathogenic microorganism infection and the host’s environmental factors such as aquaculture model, climate environment, and geography, in the process of bovine diarrheic disease have yet to be completely elucidated. The paucity of details hinders our overall comprehension of the etiology of bovine diarrheic disease.

Microbiome is a term that encompasses the collective genetic material of all microorganisms in a given environment or ecosystem, indicating the interaction between the microorganisms, their environment, and the host. At present, the identification of the human intestinal microbiome has discovered the correlation and interaction between pathogenic microorganisms and disease ecological factors, such as the host and the environment, during the occurrence of human diarrheic diseases, revealing a new pathogenesis of human diarrheic diseases. The resulting data provide a new perspective for the prevention and treatment of human diarrheic diseases (10, 11). In relevant animal research, there has been some progress in the study of gut microbiome in animals such as pigs, sheep, and even dairy cattle and yaks (12–16). By comparing the differences in gut microbiome between diarrhea and healthy animals, it has been found that there are significant differences in the enrichment of multiple microorganisms in diarrhea fecal samples. These studies provide some basic information for the study of animal gut microbiota and promote the healthy breeding of other animals and cattle. However, there is still a serious lack of information on the gut microbiome of diarrhea cattle in different disease ecological backgrounds, bacterial community which leads to an unclear pathogenesis of the diarrheic diseases that frequently occur in cattle. This leads to the poor prevention and control effect of cattle diarrhea (8, 9, 17), which has become a common scientific problem.

In order to reveal the intestinal microbiome and its characteristics of cattle suffering from diarrhea, and explore the key disease ecological risk factors of cattle diarrhea, a total of 1,016 diarrheic samples and 104 healthy samples were randomly collected from 58 farms in Heilongjiang Province of China. The virome and bacterial community of the collected samples were identified and analyzed using metagenomic technology. After examining the key ecological risk factors of bovine diarrheic disease, the etiological characteristics of the common bovine enteroviruses were revealed, including their prevalence, mixed infection, synergistic pathogenicity, genetic evolution, and cross-species transmission. This study aims to uncover novel ecological risk factors of bovine diarrheic disease by examining the pathogenic microorganism-host-environmental disease ecology, thereby providing a theoretical basis for comprehensive prevention and control strategies of bovine diarrheic disease, as well as a reference for the research of human and other animal diarrheic diseases.

## RESULTS

### Overview of virome and bacterial community

**Fig 1:**
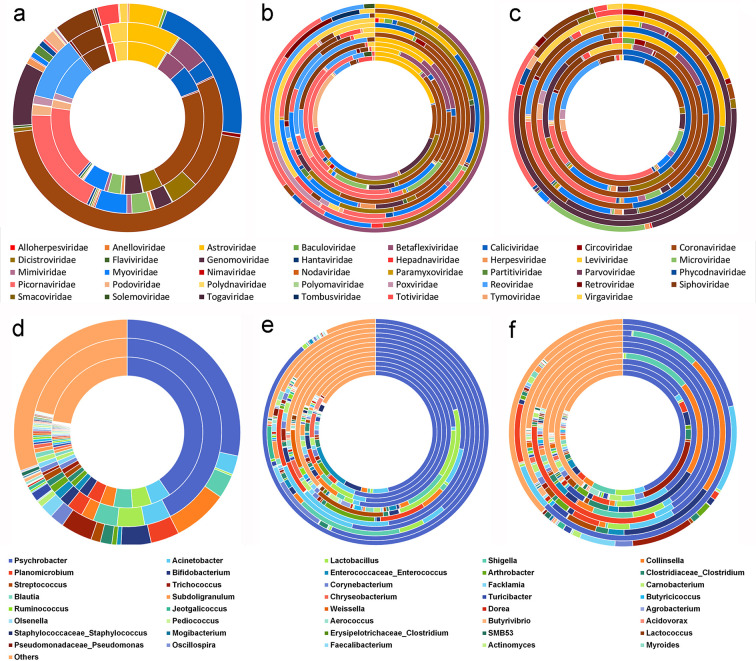
The composition of virome at the family level and bacterial community at the genus level detected in cattle fecal samples. (**a**) Composition of virome in the whole, diarrhea, and healthy samples (from the inner to the outer circle). (**b**) Composition of virome in cattle fecal samples in 12 cities (regions) of Heilongjiang Province. From the inner circle to the outer circle were Suihua, Shuangyashan, Heihe, Harbin, Qiqihar, Mudanjiang, Daxinganling, Hegang, Yichun, Jixi, Jiamusi, and Daqing, respectively. (**c**) Composition of the virome in cattle fecal samples under various factors. From the inner circle to the outer circle were diarrhea-intensive, health-intensive, diarrhea-non-intensive, health-non-intensive, diarrhea-Holstein, health-Holstein, diarrhea-Simmental, health-Simmental, diarrhea-Angus, and health-Angus. (**d**) Composition of bacterial community in the whole, diarrhea, and healthy samples (from the inner to the outer circle). (**e**) Composition of bacterial community in cattle in 12 cities (regions) of Heilongjiang Province. Inner to outer is the same as panel **b**. (**f**) Composition of bacterial community in cattle fecal samples under various factors. Inner to outer is the same as panel **c**.

1,120 fecal samples that were randomly collected from 58 farms were pooled into 72 samples. The 72 pooled samples included the 62 pooled samples from 1,016 diarrheic cattle and the 10 pooled samples from 104 healthy cattle. The detailed information regarding the pooled samples is available in Table S1. Disease ecology information regarding 1,016 diarrheic samples and 104 healthy samples is shown in Table 1. The virome of the 72 pooled samples was identified by using the Illumina NovaSeq 6000 system. Results indicated that, of the 70 samples that were successfully sequenced, a total of 39 virus families, 86 virus genera, and 110 virus species were identified. In diarrheic samples, the number of virus families, genera, and species identified was 38, 82, and 102, respectively, while the healthy samples revealed 25 virus families, 37 virus genera, and 41 virus species. These data were shown in Table S2. Out of 70 mixed sequencing samples, a total of 322 complete or nearly complete viral genomes were identified; 208 of them (64.6%) belonged to RNA viruses and the remaining 114 (35.4%) were classified as DNA viruses. A total of 191 bovine diarrhea-associated viruses, 79 other eukaryotic viruses, and 52 *Microviridae* have been identified. At the family level, a total of 72 whole-genome sequences of *Astroviridae*, 53 of *Coronaviridae*, 52 of *Microviridae*, 51 of *Genomoviridae*, 36 of *Caliciviridae*, 32 of *Picornaviridae*, 11 of *Circoviridae*, 9 of *Dicistroviridae*, 5 of *Reoviridae*, and 1 of *Flaviviridae* were identified, respectively (Table S3). The abundance of viruses at the family level is shown in Fig. 1. A total of 39 virus families were identified in all samples, among which 38 virus families were identified in diarrhea fecal samples; the top 10 viral families with the highest abundance were *Coronaviridae*, *Picornaviridae*, *Reoviridae*, *Astroviridae*, *Betaflexiviridae*, *Siphoviridae*, *Myoviridae*, *Dicistroviridae*, *Microviridae*, and *Virgaviridae*. Twenty-five viral families were detected in the health samples, and the top 10 viral families with the highest abundance were *Coronaviridae*, *Caliciviridae*, *Genomoviridae*, *Siphoviridae*, *Astroviridae*, *Totiviridae*, *Podoviridae*, *Partiviridae*, *Virgaviridae*, and *Herpesviridae* (Fig. 1a).

**TABLE 1:** Summary table of sample collection and classification

	Factors	Classification	Number of cattle farms	Number of cattle
Host factors	Clinical status	Diarrhea	58	1,016
		Healthy	18	104
	Age	Calf	46	555
		Adult	34	565
	Cattle type	Dairy cow	34	562
		Beef cattle	24	558
	Gender	Male	24	109
		Female	57	1,011
Environmental factors	Aquaculture model	Intensive	45	687
		Non-intensive	15	433
	Geography	East region	13	314
		West region	18	193
		South region	7	164
		North region	20	449

Analysis of the virome at the family level in diarrheic and healthy fecal samples from different cities (regions), aquaculture models, and breeds revealed varying abundances and compositions. Among the 12 cities (regions) in Heilongjiang Province, cattle fecal samples from Heihe City revealed the most abundant virome composition, with a total of 25 viral families identified. *Coronaviridae*, *Picornaviridae*, *Betaflexiviridae*, *Reoviridae*, and *Microviridae* were the top five viral families with the highest abundance. Cattle fecal samples from Daqing City revealed the simplest virome composition, with a total of 7 viral families identified. *Betaflexiviridae*, *Picornaviridae*, *Astroviridae*, *Reoviridae*, and *Coronaviridae* were the top five viral families with the highest abundance (Fig. 1b). Results of studying the virome composition in different aquaculture models indicated that 31 viral families were detected in diarrhea fecal samples in intensive farming conditions, the *Picornaviridae*, *Reoviridae*, *Coronaviridae*, *Microviridae*, and *Caliciviridae* were the top five viral families with the highest abundance. Two viral families were detected in healthy fecal samples, including *Caliciviridae* and *Coronaviridae*. Thirty-two viral families were detected in diarrhea fecal samples in non-intensive farming conditions, the *Coronaviridae*, *Dicistroviridae*, *Myoviridae*, *Picornaviridae*, and *Siphoviridae* were the top five viral families with the highest abundance. Seven viral families were detected in the healthy samples, the *Coronaviridae*, *Genomoviridae*, *Siphoviridae*, *Baculoviridae*, and *Dicistroviridae* were the top five viral families with the highest abundance. Results of studying the virome composition in different cattle breeds indicated that 34 viral families were detected in diarrhea fecal samples from Simmental cattle, the *Coronaviridae*, *Dicistroviridae*, *Picornaviridae*, *Myoviridae*, and *Siphoviridae* were the top five viral families with the highest abundance. Six viral families were detected in healthy fecal samples, the *Astroviridae*, *Siphoviridae*, *Genomoviridae*, *Virgaviridae*, and *Baculoviridae* were the top five viral families with the highest abundance. Twenty viral families were detected in diarrhea fecal samples from Angus cattle, the *Genomoviridae*, *Picornaviridae*, *Astroviridae*, *Microviridae*, and *Siphoviridae* were the top five viral families with the highest abundance. Nine viral families were detected in healthy fecal samples, the *Genomoviridae*, *Coronaviridae*, *Siphoviridae*, *Totiviridae*, and *Circoviridae* were the top five viral families with the highest abundance. Twenty-six viral families were detected in diarrhea fecal samples from Holstein cattle, the *Picornaviridae*, *Coronaviridae*, *Reoviridae*, *Astroviridae*, and *Betaflexiviridae* were the top five viral families with the highest abundance. Three viral families were detected in healthy fecal samples, including *Coronaviridae*, *Caliciviridae*, and *Siphoviridae* (Fig. 1c).

Seventy mixed sequencing samples were identified and analyzed for 16S rRNA diversity of bacteria using the Illumina Miseq sequencing platform. The results showed that a total of 39 bacterial phyla, 444 bacterial families, 1,011 bacterial genera, and 1,421 bacterial species were identified. Of these, 435 bacterial families, 977 bacterial genera, and 1,373 bacterial species were identified in diarrhea fecal samples, and 172 bacterial families, 395 bacterial genera, and 531 bacterial species were identified in healthy samples. Results are shown in Table S4. At the bacterial genus level, the top 40 bacterial genera with abundance are shown in Fig. 1d; among them, the top 10 bacterial genera with the highest abundance in diarrhea fecal samples were *Psychrobacter*, *Lactobacillus*, *Acinetobacter*, *Shigella*, *Planomicrobium*, *Collinsella*, *Bifidobacterium*, *Enterococcaceae*_*Enterococcus*, *Arthrobacter*, and *Streptococcus*. The top 10 bacterial genera with the highest abundance in healthy samples were *Psychrobacter*, *Collinsella*, *Trichococcus*, *Bifidobacterium*, *Planomicrobium*, *Shigella*, *Acinetobacter*, *Corynebacterium*, *Facklamia*, and *Clostridiaceae*_*Clostridium*.

Analysis of the bacterial community at the genus level in diarrheic and healthy fecal samples from different cities (regions), aquaculture models, and breeds revealed varying abundances and compositions. Among the 12 cities (regions) in Heilongjiang Province, cattle fecal samples from Qiqihar City revealed the most abundant bacterial community composition, with a total of 720 bacterial genera identified. *Psychrobacter*, *Lactobacillus*, *Shigella*, *Pediococcus*, and *Agrobacterium* were the top five bacterial genera with the highest abundance. Cattle fecal samples from Jiamusi City revealed the simplest bacterial community composition, with a total of 140 bacterial genera identified. *Psychrobacter*, *Corynebacterium*, *Jeotgalicoccus*, *Facklamia*, and *Clostridiaceae*_*Clostridium* were the top five bacterial genera with the highest abundance (Fig. 1e). Results of studying the bacterial community composition in aquaculture model indicated that 845 bacterial genera were detected in diarrhea fecal samples in intensive farming conditions, and the *Psychrobacter*, *Shigella*, *Lactobacillus*, *Collinsella*, and *Acinetobacter* were the top five bacterial genera with the highest abundance. Two hundred sixteen bacterial genera were detected in the healthy fecal samples, the *Trichococcus*, *Psychrobacter*, *Bifidobacterium*, *Facklamia*, and *Carnobacterium* were the top five bacterial genera with the highest abundance. Six hundred ninety-three bacterial genera were detected in diarrhea fecal samples under non-intensive farming conditions, the *Psychrobacter*, *Acinetobacter*, *Lactobacillus*, *Planomicrobium*, and *Arthrobacter* were the top five bacterial genera with the highest abundance. The 90 bacterial genera were detected in healthy samples, the *Collinsella*, *Bifidobacterium*, *Shigella*, *Clostridiaceae*_*Clostridium*, and *Turicibacter* were the top five bacterial genera with the highest abundance. Results of studying the bacterial community composition in different cattle breeds indicated that 820 bacterial genera were detected in diarrhea fecal samples from Holstein cattle, the *Psychrobacter*, *Shigella*, *Lactobacillus*, *Collinsella*, and *Enterococcaceae*_*Enterococcus* were the top five bacterial genera with the highest abundance. Ninety-six bacterial genera were detected in healthy fecal samples, the *Psychrobacter*, *Planomicrobium*, *Acinetobacter*, *Carnobacterium*, and *Clostridiaceae*_*Clostridium* were the top five bacterial genera with the highest abundance. Six hundred ninety-four bacterial genera were detected in diarrhea fecal samples from Simmental cattle, and the *Psychrobacter*, *Acinetobacter*, *Planomicrobium*, *Lactobacillus*, and *Arthrobacter* were the top five bacterial genera with the highest abundance. One hundred eleven bacterial genera were detected in healthy fecal samples, the *Collinsella*, *Bifidobacterium*, *Shigella*, *Streptococcus*, and *Turicibacter* were the top five bacterial genera with the highest abundance. Two hundred ninety-six bacterial genera were detected in diarrhea fecal samples from Angus cattle, the *Psychrobacter*, *Planomicrobium*, *Acinetobacter*, *Carnobacterium*, and *Subdoligranulum* were the top five bacterial genera with the highest abundance. One hundred ninety-one bacterial genera were detected in healthy fecal samples, the *Psychrobacter*, *Acinetobacter*, *Trichococcus*, *Facklamia*, and *Corynebacterium* were the top five bacterial genera with the highest abundance (Fig. 1f).

### Fecal virome-bacterial community analysis of the diarrhea-affected cattle with various environment and host conditions

**Fig 2:**
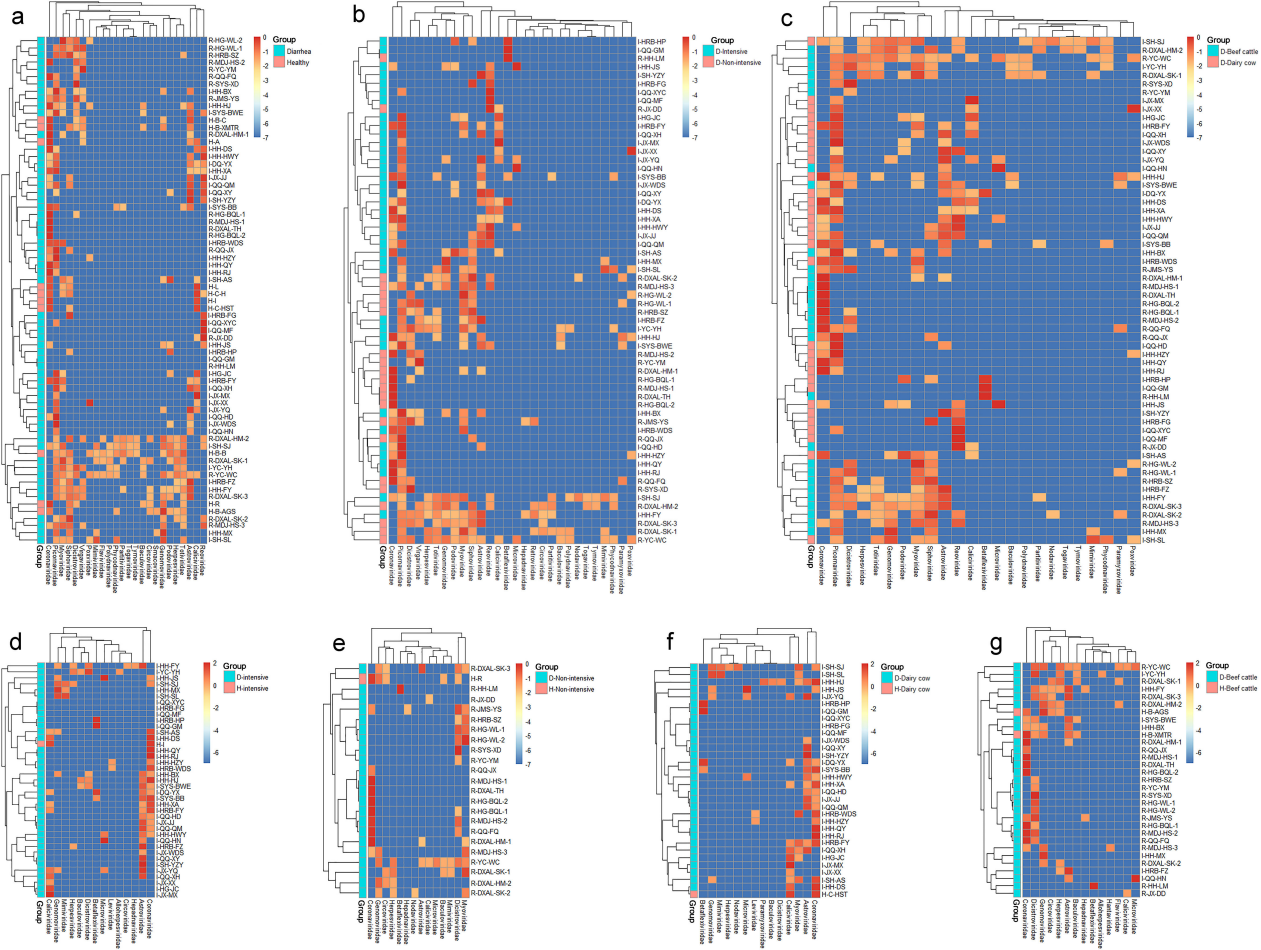
Heatmap abundance analysis of virome at the family level in cattle fecal samples. (**a**) Heatmap abundance analysis of virome in diarrheic and healthy cattle fecal samples. (**b**) Heatmap abundance analysis of virome in diarrheal fecal samples under intensive and non-intensive farming. (**c**) Heatmap abundance analysis of virome in diarrheal fecal samples from dairy cows and beef cattle. (**d**) Heatmap abundance analysis of virome in diarrheic and healthy cattle fecal samples under intensive farming. (**e**) Heatmap abundance analysis of virome in diarrheic and healthy cattle fecal samples under non-intensive farming. (**f**) Heatmap abundance analysis of virome in diarrheic- and healthy-dairy cow fecal samples. (**g**) Heatmap abundance analysis of virome in diarrheic- and healthy-beef cattle fecal samples.

In order to study the fecal virome-bacterial community characteristics of the diarrhea-affected cattle with various environment and host conditions, heatmap analysis was performed. Heatmap analysis of the virome at the family level indicated that the different distribution patterns in different clinical statuses, aquaculture models, and types of cattle. Under different clinical statuses, the *Reoviridae*, *Picornaviridae*, *Genomoviridae*, *Coronaviridae*, *Circoviridae*, and *Astroviridae* in diarrhea fecal samples showed high abundance, and *Coronaviridae* also showed high abundance in healthy samples, and only one sample found *Reoviridae* (Fig. 2a). Under different aquaculture models, the *Circoviridae*, *Coronaviridae*, *Genomoviridae*, *Dicistroviridae*, and *Myoviridae* showed high abundance in cattle diarrhea fecal samples under non-intensive aquaculture model (Fig. 2b and e). *Coronaviridae* and *Astroviridae* showed high abundance in several samples of diarrhea fecal samples from intensively farmed cattle, while only *Coronaviridae* and *Caliciviridae* were observed in healthy fecal samples (Fig. 2d). Of the various types of cattle, *Picornaviridae*, *Coronaviridae*, *Reoviridae*, *Astroviridae* and *Caliciviridae* showed high abundance in the fecal samples of dairy cow with diarrhea, while only *Coronaviridae* and *Caliciviridae* were found in healthy samples (Fig. 2c and f). In the fecal samples of beef cattle with diarrhea, *Coronaviridae* and *Dicistroviridae* showed a high abundance in several of the diarrhea samples, and in healthy dairy cattle fecal samples, *Coronaviridae* and *Dicistroviridae* also showed a high abundance (Fig. 2g).

**Fig 3:**
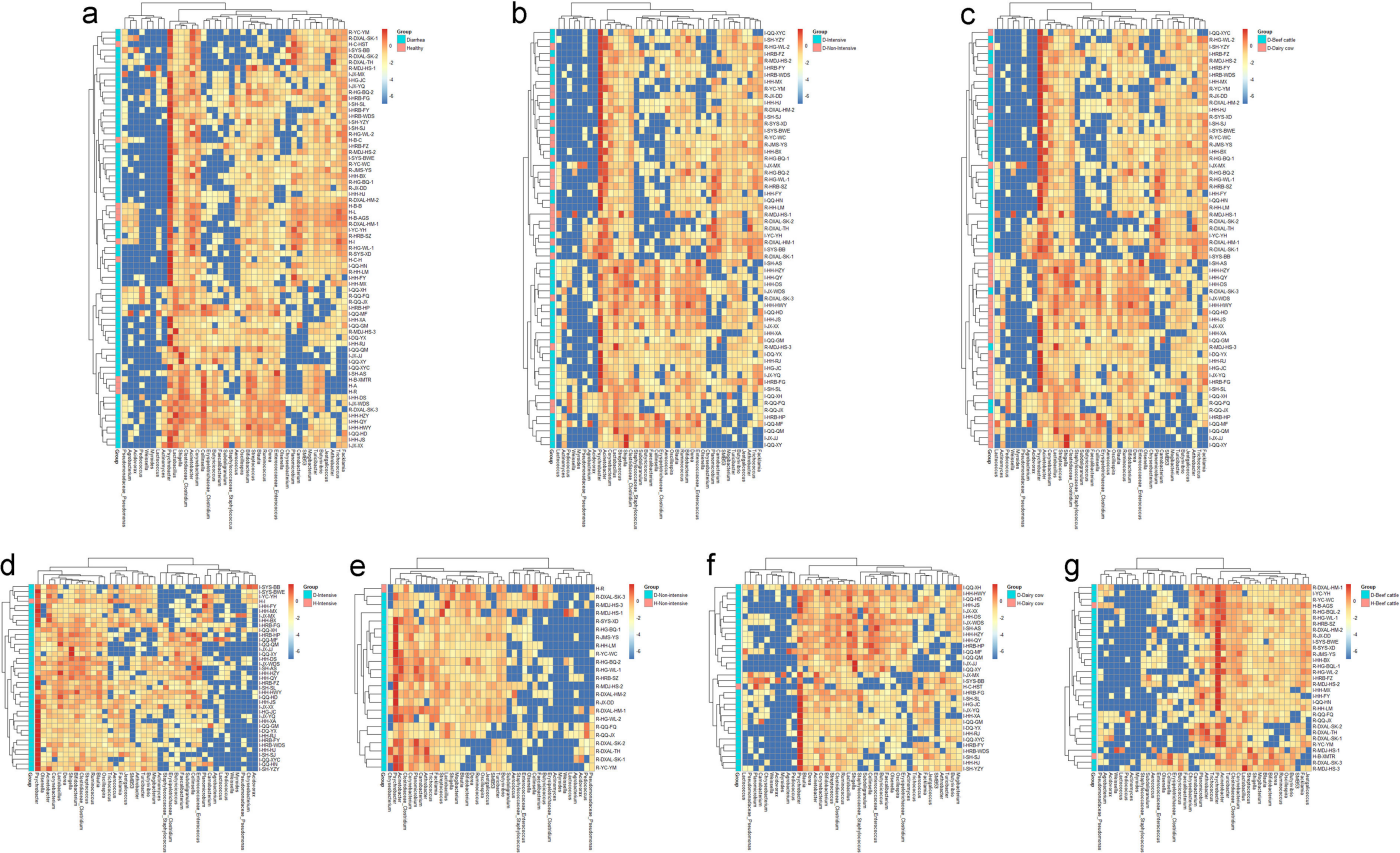
Heatmap abundance analysis of bacterial community at the genus level in cattle fecal samples. (**a**) Heatmap abundance analysis of the bacterial community in diarrheic and healthy cattle fecal samples. (**b**) Heatmap abundance analysis of the bacterial community in cattle diarrheic fecal samples under intensive and non-intensive farming. (**c**) Heatmap abundance analysis of the bacterial community in dairy cow and beef cattle diarrheic fecal samples. (**d**) Heatmap abundance analysis of bacterial community in diarrheic and healthy cattle fecal samples under intensive farming. (**e**) Heatmap abundance analysis of the bacterial community in diarrheic and healthy cattle fecal samples under non-intensive farming. (**f**) Heatmap abundance analysis of the bacterial community in diarrheic- and healthy-dairy cow fecal samples. (**g**) Heatmap abundance analysis of the bacterial community in diarrheic and healthy beef cattle fecal samples.

Heatmap analysis of the bacterial community at the genus level indicated that the different distribution patterns in different clinical statuses, aquaculture models, and types of cattle. Under different clinical statuses, the *Lactobacillus*, *Shigella*, and *Enterococcaceae*_*Enterococcus* in several diarrhea fecal samples showed high abundance, while *Collinsella* and *Bifidobacterium* showed high abundance in several healthy samples (Fig. 3a). Under different aquaculture models, the *Shigella*, *Subdoligranulum and Enterococcaceae*_*Enterococcus* showed high abundance in cattle diarrhea fecal samples under intensive aquaculture model, and *Acinetobacter*, *Planomicrobium*, and *Streptococcus* showed high abundance in cattle diarrhea fecal samples under non-intensive aquaculture model (Fig. 3b and d). *Psychrobacter*, *Acinetobacter*, *Planomicrobium*, *Arthrobacter*, and *Carnobacterium* showed high abundance in multiple samples of diarrhea fecal samples from non-intensive aquaculture model. *Shigella*, *Collinsella*, and *Bifidobacterium* showed high abundance in both diarrheic and healthy fecal samples (Fig. 3e). Of the various types of cattle, *Shigella*, *Enterococcaceae*_*Enterococcus*, *Subdoligranulum*, and *Erysipelotrichaceae*_*Clostridium* showed high abundance in dairy cattle diarrhea fecal samples. *Planomicrobium* and *SMB53* showed high abundance in beef cattle diarrhea feces, while *Shigella*, *Collinsella*, *Enterococcaceae*_*Enterococcus*, *Subdoligranulum*, and *Butyricicoccus* only exist in dairy cattle diarrhea fecal samples (Fig. 3c and f). *Planomicrobium*, *Carnobacterium*, and *Acinetobacter* showed high abundance in the fecal samples of beef cattle with diarrhea. Among the healthy fecal samples of beef cattle, *Trichococcus*, *Psychrobacter*, *Actinomyces*, *Collinsella*, *Shigella*, and *Bifidobacterium* showed high abundance in several samples (Fig. 3g).

**Fig 4:**
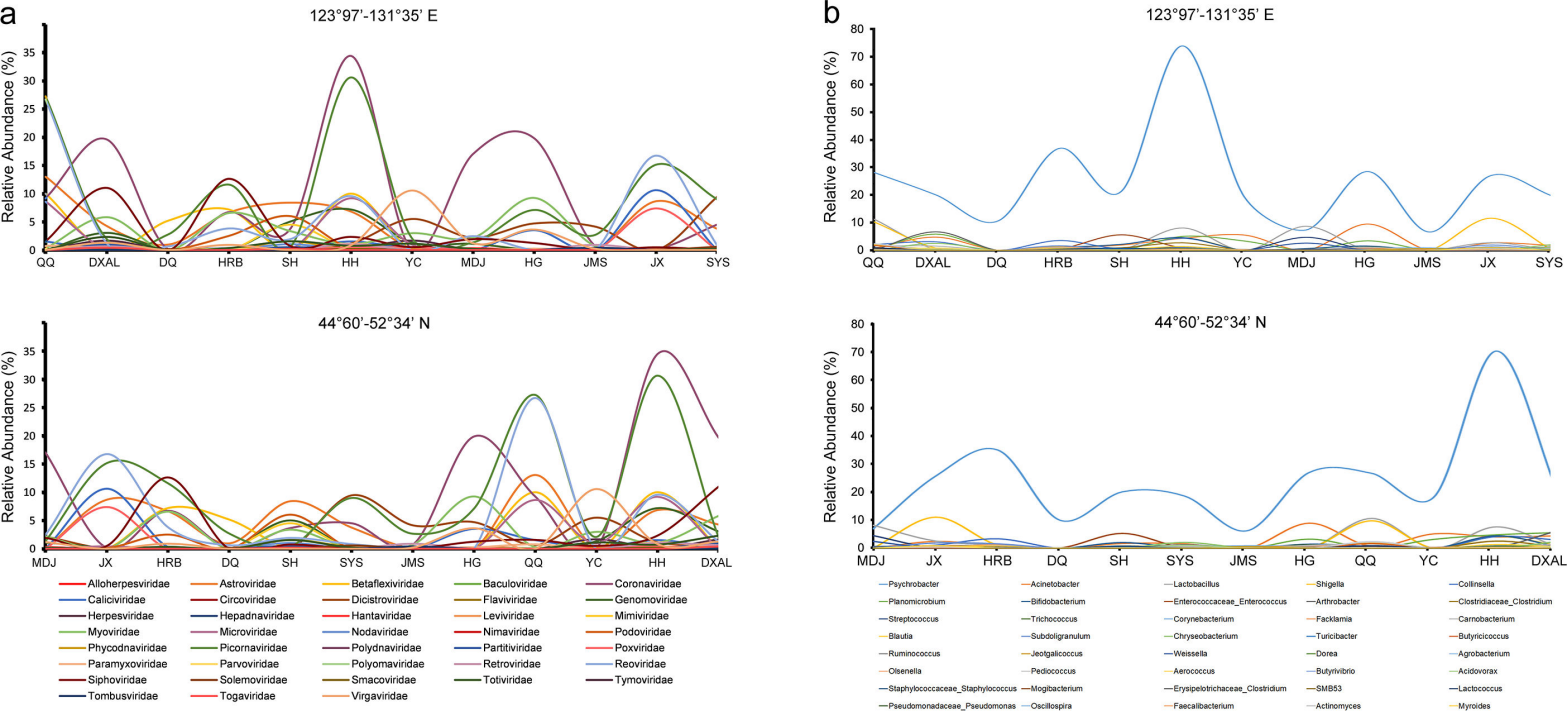
Relative abundance of virome at the family level and bacterial community at the genus level with longitude and latitude variations in cattle diarrheic fecal samples. (**a**) Relative abundance of virome with longitude and latitude variations. (**b**) Relative abundance of bacterial community with longitude and latitude variations.

Variations in the relative abundance of virome at the family level and bacterial community at the genus level can be observed in cattle diarrheic fecal samples depending on the longitude and latitude. The analysis of different geographical locations showed that the abundance of *Reoviridae* increased with the increase of longitude in virome analysis, with the highest in Jixi City (longitude 130.97°E). The abundance of *Polydnaviridae*, *Microviridae*, and *Picornaviridae* increased with the increase of latitude, with the highest in Heihe City (latitude 50.24°N) (Fig. 4a). Bacterial community analysis showed that the abundance of *Psychrobacter* was high in all cities (regions), with the highest in Heihe City (latitude 50.24°N, longitude 127.49°E). The abundance of *Shigella* was high in Jixi City (latitude 45.30°N, longitude 130.97°E) (Fig. 4b).

### Correlation analysis of the fecal virome-bacterial community of cattle affected by diarrhea with various environmental and host conditions

**Fig 5:**
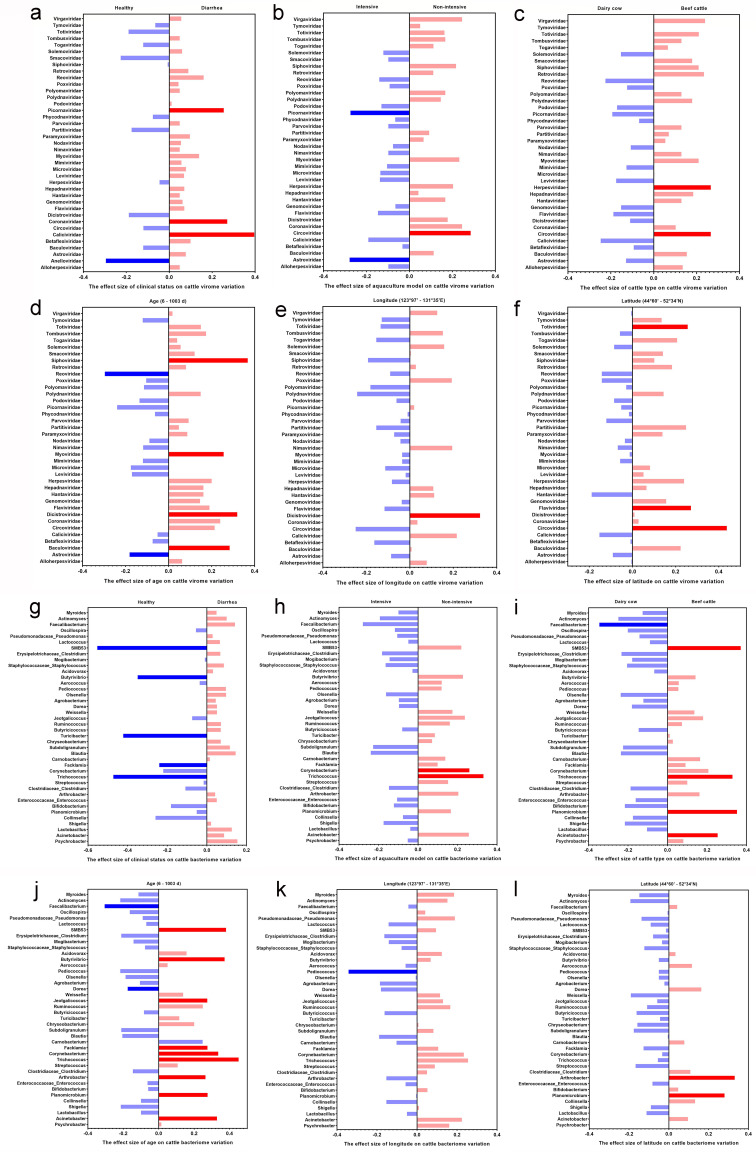
Effect size of various factors on the abundance of virome at the family level and bacterial community at the genus level in cattle fecal samples. (**a**) The effect size of clinical status on cattle virome variation. (**b**) The effect size of aquaculture model on cattle virome variation. (**c**) The effect size of cattle type on cattle virome variation. (**d**) The effect size of age on cattle virome variation. (**e**) The effect size of longitude on cattle virome variation. (**f**) The effect size of latitude on cattle virome variation. (**g**) The effect size of clinical status on cattle bacterial community variation. (**h**) The effect size of aquaculture model on cattle bacterial community variation. (**i**) The effect size of cattle type on cattle bacterial community variation. (**j**) The effect size of age on cattle bacterial community variation. (**k**) The effect size of longitude on cattle bacterial community variation. (**l**) The effect size of latitude on cattle bacterial community variation.

In order to further reveal the viruses or bacteria that were correlated with diarrhea-affected cattle with various environment and host conditions, a correlation analysis of the virome and bacterial community was performed in fecal samples from diarrhea-affected cattle by using regression analysis, a correlation heatmap, a chord diagram analysis, and a network association diagram. Binary logistic regression and multiple linear regression were used to analyze the correlation between virome and bacterial community and different host factors and environmental factors. The virome regression analysis showed that, under different host factors and environmental factors, there were significant differences in the abundance of virome at the family level in cattle fecal samples. Under different clinical statuses, the abundance of *Picornaviridae*, *Coronaviridae*, *Caliciviridae*, and *Anelloviridae* was significantly correlated with the clinical statuses (*P <* 0.05). Among them, the abundance of *Picornaviridae*, *Coronaviridae*, and *Caliciviridae* was significantly correlated with diarrhea (*P <* 0.05) (Fig. 5a). Under different aquaculture models, the abundance of *Picornaviridae* and *Astroviridae* in diarrhea fecal samples was significantly correlated with intensive aquaculture model (*P <* 0.05), and the abundance of *Circoviridae* was significantly correlated with non-intensive aquaculture model (*P <* 0.05) (Fig. 5b). In different types of cattle, the abundance of *Herpesviridae* and *Circoviridae* was significantly correlated with that of beef cattle (*P <* 0.05) (Fig. 5c). Under different age factors of cattle, the abundance of *Astroviridae*, *Reoviridae*, *Siphoviridae*, *Myoviridae*, *Dicistroviridae*, and *Baculoviridae* was significantly correlated with the age of cattle (*P <* 0.05), of which, the abundance of *Astroviridae* and *Reoviridae* was significantly correlated with calves (*P <* 0.05) (Fig. 5d). In different geographical locations, the abundance of *Dicistroviridae* was significantly correlated with longitude (*P <* 0.05) (Fig. 5e). The abundance of *Totiviridae*, *Flaviviridae*, and *Circoviridae* was significantly correlated with latitude (*P <* 0.05) (Fig. 5f). The bacterial community regression analysis showed that, under different host factors and environmental factors, there were also significant differences and abundance of bacterial community at the genus level in cattle fecal samples. Under different clinical statuses, the abundance of *SMB53*, *Butyrivibrio*, *Facklamia*, *Trichococcus*, and *Turicibacter* was significantly correlated with health (*P <* 0.05) (Fig. 5g). In different aquaculture models, the abundance of *Corynebacterium* and *Trichococcus* in diarrhea fecal samples was significantly correlated with the non-intensive aquaculture model (*P <* 0.05) (Fig. 5h). Among the different cattle types, the abundance of *Faecalibacterium*, *SMB53*, *Trichococcus*, *Planomicrobium*, and *Acinetobacter* was significantly correlated with the cattle type (*P <* 0.05), and the abundance of *Faecalibacterium* was significantly correlated with the dairy cow (*P <* 0.05). The abundance of *SMB53*, *Trichococcus*, *Planomicrobium*, and *Acinetobacter* was significantly correlated with beef cattle (*P <* 0.05) (Fig. 5i). Under different age factors of cattle, the abundance of *Faecalibacterium* and *Dorea* was significantly correlated with calves (*P <* 0.05), and the abundance of *SMB53*, *Butyrivibrio*, *Jeotgalicoccus*, *Facklamia*, *Corynebacterium*, *Trichococcus*, *Arthrobacter*, *Planomicrobium*, and *Acinetobacter* was significantly correlated with adult cattle (*P <* 0.05) (Fig. 5j). In different geographical locations, the abundance of *Pediococcus* was significantly correlated with longitude (*P <* 0.05) (Fig. 5k). The abundance of *Arthrobacter* and *Planomicrobium* was significantly correlated with latitude (*P <* 0.05) (Fig. 5l).

**Fig 6:**
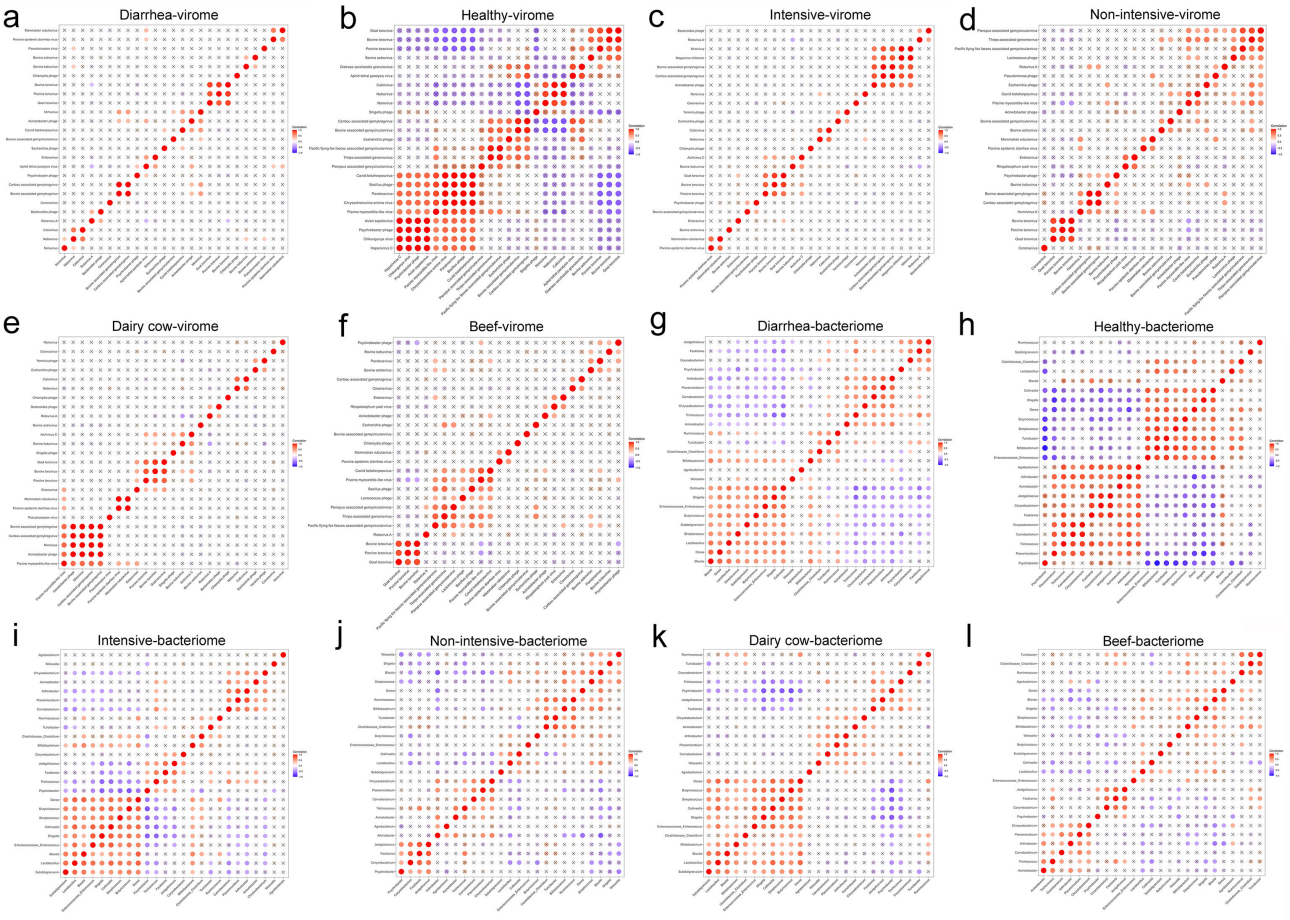
Intra-kingdom correlation analysis in the virome at species level and bacterial community at the genus level under various factors. (**a–f**) Intra-kingdom correlation analysis of virome in cattle fecal samples under various factors. (**c–f**) Intra-kingdom correlation analysis of virome in cattle diarrheic fecal samples under various factors. (**g–l**) Intra-kingdom correlation analysis of bacterial community in cattle fecal samples under various factors. (**i–l**) Intra-kingdom correlation analysis of bacterial community in diarrheic fecal samples from cattle under various factors. Statistical analysis was performed using Spearman correlation analysis.

In order to analyze the in-boundary correlation virome at the family level and the bacterial community at the genus level under different host and environmental factors, Spearman correlation analysis was used to display the correlation matrix. The results of the correlation matrix of viromes showed that viruses exhibit different interactions with each other under various host and environmental factors. Under different clinical statuses, in diarrhea fecal samples, bovine kobuvirus (BKV) interacted synergistically with nebovirus and bovine astrovirus (BoAstV) (*P <* 0.05), and nebovirus interacted with calicivirus and norovirus (*P <* 0.05). The interaction between bovine torovirus (BToV) and rotavirus A was antagonistic (*P <* 0.05) (Fig. 6a). In healthy fecal samples, nebovirus and norovirus interacted synergistically (*P <* 0.05) (Fig. 6b). In different aquaculture models, BToV interacted synergistically with aichivirus C, and nebovirus interacted synergistically with norovirus (*P <* 0.05) in the fecal samples of intensive cattle diarrhea (Fig. 6c). However, under the non-intensive aquaculture model, hunnivirus A and bovine-associated gemykrogvirus interacted synergistically with each other (*P <* 0.05) (Fig. 6d). In different types of cattle, enterovirus interacted synergistically with BToV in dairy cow diarrhea fecal samples (*P <* 0.05). BToV and BKV interacted synergistically (*P <* 0.05). The interaction between nebovirus and calicivirus was synergistic (*P <* 0.05) (Fig. 6e). In the fecal samples of beef cattle diarrhea, BoAstV interacted synergistically with BKV and pandoravirus (*P <* 0.05) (Fig. 6f). The results of the correlation matrix of the bacterial community showed that bacteria exhibit different interactions with each other under various host and environmental factors. Under different clinical statuses, in diarrhea fecal samples, *Clostridiaceae_Clostridium* interacted synergistically with *Blautia*, *Dorea*, *Butyricicoccus*, *Bifidobacterium*, *Turcicactor*, *Ruminooccus*, and *Corynebacterium* (*P <* 0.05). A variety of pathogenic bacteria and probiotics, including *Butyricicoccus*, *Streptococcus*, *Collinsella*, *Shigella*, *Enterococcaceae_Enterococcus*, *Dorea*, *Bifidobacterium*, and *Turicibacter*, interacted synergistically with each other (*P <* 0.05) (Fig. 6g). In healthy fecal samples, *Clostridiaceae_Clostridium* interacted synergistically with *Dorea*, *Bifidobacterium*, and *Turicibacter* (*P <* 0.05). *Collinsella* interacted synergistically with *Shigella*, *Enterocaceae_Enterococcus*, *Butyriciccus*, *Streptococcus*, *Dorea*, *Bifidobacterium*, *and Turicibacter* (*P <* 0.05). *Enterococcaceae_Enterococcus* interacted synergistically with *Collinsella*, *Shigella*, *Butyricicoccus*, *Streptooccus*, *Dorea*, *Bifidobacterium*, and *Turicibacter* (*P <* 0.05) (Fig. 6h). In different aquaculture models, the fecal samples of intensively farmed cattle with diarrhea showed that *Clostridiaceae*_*Clostridium* interacted synergistically with *Blautia*, *Butyricicoccus*, *Dorea*, *Bifidobacterium*, *Turicibacter*, *Ruminococcus*, and *Streptococcus* (*P* < 0.05). The interaction between *Shigella*, *Enterococcaceae*_*Enterococcus*, *Bifidobacterium*, *Subdoligranulum*, *Lactobacillus*, *Blautia*, *Collinsella*, *Streptococcus*, *Butyricicoccus*, and *Dorea* (*P <* 0.05) (Fig. 6i). In the fecal samples of diarrhea from non-intensively farmed cattle, *Shigella* interacted synergistically with *Streptococcus*, *Blautia*, and *Weissella* (*P <* 0.05) and antagonized with *Facklamia* and *Trichococcus* (*P <* 0.05). *Clostridiaceae_Clostridium* interacted synergistically with *Turicibacter*, *Bifidobacterium*, and *Ruminocus* (*P <* 0.05), while it interacted antagonistically with *Collinsella* (*P <* 0.05). *Enterococcaceae_Enterococcus* and *Bifidobaterium* interacted synergistically (*P <* 0.05). A variety of probiotics, including *Ruminococcus*, *Bifidobacterium*, *Lactobacillus*, and *Butyricicoccus*, interact synergistically with each other (*P <* 0.05) (Fig. 6j). In different types of cattle, in dairy cow diarrhea fecal samples, *Enterococcaceae*_*Enterococcus* and *Shigella*, *Subdoligranulum*, *Butyricicoccus*, *Lactobacillus*, *Collinsella*, Streptococcus, *Butyricicoccus*, and *Dorea* interacted synergistically (*P <* 0.05). *Clostridiaceae*_*Clostridium* and *Blautia*, *Bifidobacterium*, *Streptococcus*, *Butyricicoccus*, *Dorea*, *Turicibacter*, *Ruminococcus* interacted synergistically (*P <* 0.05) (Fig. 6k). *Clostridiaceae*_*Clostridium* interacted synergistically with *Facklamia*, *Jeotgalicoccus*, *Bifidobacterium*, *Dorea*, *Ruminococcus*, *Turicibacter* (*P <* 0.05), but antagonized with *Collinsella* (*P <* 0.05). *Shigella* interacted synergistically with *Lactobacillus*, *Weissella*, *Blautia*, and *Dorea* (*P <* 0.05), and antagonized with *Trichococcus* and *Facklamia* (*P <* 0.05). *Enterococcaceae_Enterococcus* interacted with *Lactobacillus* and *Bifidobacterium* (*P <* 0.05) (Fig. 6l).

**Fig 7:**
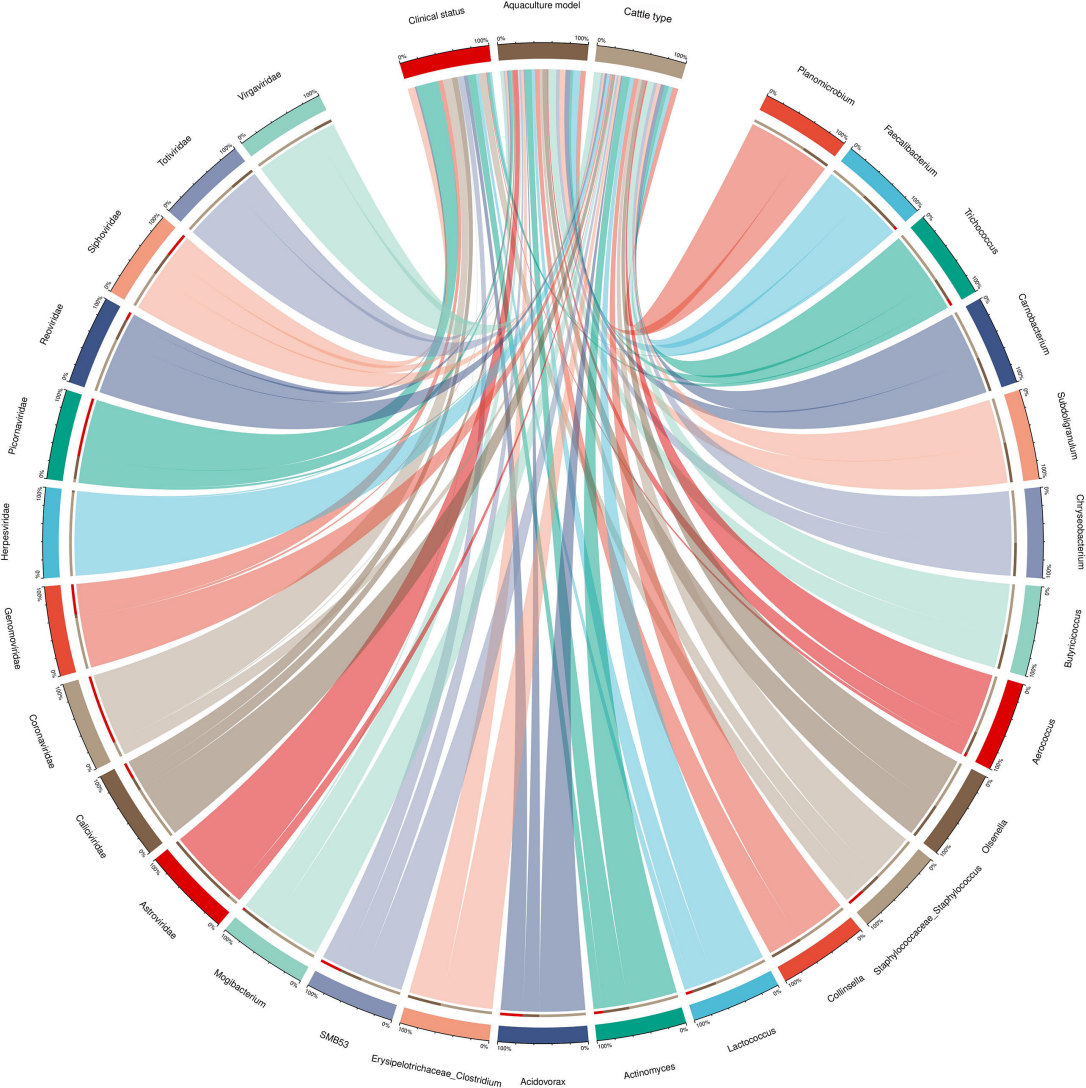
Correlation analysis of virome at the family level and bacterial community at the genus level in cattle diarrheic fecal samples with various factors.

In order to comprehensively identify the correlation between virome, bacterial community and different risk factors. Chi-squared test analysis was carried out between the virome, bacterial community and clinical status, aquaculture model, type of cattle in diarrhea fecal samples. The results showed that there was a significant correlation between the level of virome, bacterial community, and different factors. The abundance of *Coronaviridae* and *Piconaviridae* was significantly correlated with clinical status (*P <* 0.05). The abundance of *Astroviridae*, *Picornaviridae*, *Virgaviridae*, *Collinsella*, *Butyricicoccus*, *Planomicrobium*, *Trichococcus*, *Carnobacterium*, *Subdoligranulum*, *Chryseobacterium*, *Olsenella*, *Aerococcus*, *Lactococcus*, *Staphylococcaceae*_*Staphylococcus*, *Erysipelotrichaceae*_*Clostridium*, *Faecalibacterium*, *Actinomyces*, and *Actinomyces* was significantly correlated with the aquaculture model (*P <* 0.05). The abundance of *Caliciviridae*, *Genomoviridae*, *Herpesviridae*, *Reoviridae*, *Siphoviridae*, *Totiviridae*, *Virgaviridae*, *Collinsella*, *Butyricicoccus*, *Planomicrobium*, *Trichococcus*, *Carnobacterium*, *Subdoligranulum*, *Chryseobacterium*, *Olsenella*, *Aerococcus*, *Lactococcus*, *Staphylococcaceae*_*Staphylococcus*, *Erysipelotrichaceae*_*Clostridium*, *Faecalibacterium*, *Actinomyces*, *Acidovorax*, *Mogibacterium*, and *SMB53* was significantly correlated with the type of cattle (*P <* 0.05) (Fig. 7; Table S5).

**Fig 8:**
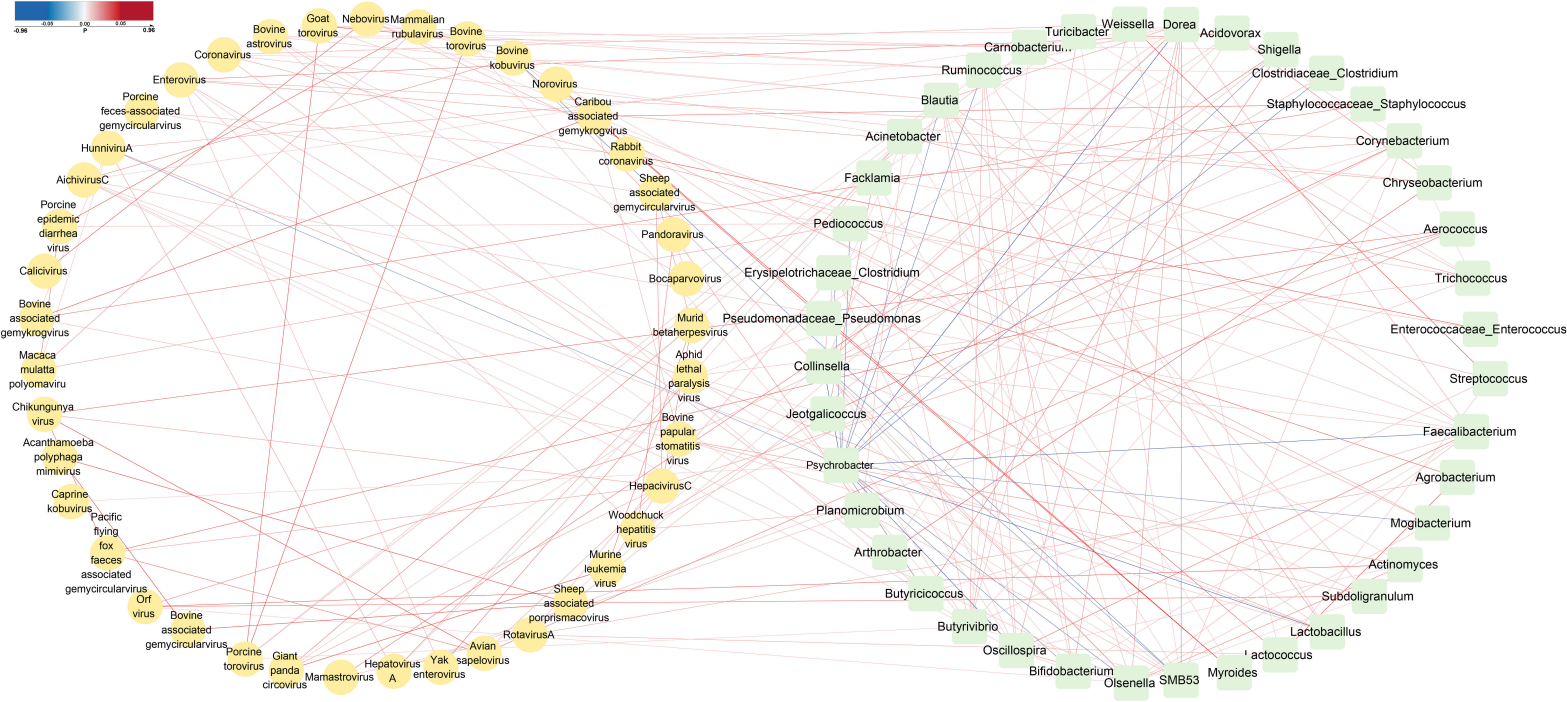
Intra-boundary and inter-boundary correlations between the virome at the species level and the bacterial community at the genus level in diarrheic fecal samples from cattle. Correlation coefficient was calculated, while statistical significance was determined for all pairwise comparisons. Only statistically significant correlations with |correlation coefficient| > 0.26 were plotted. The correlation network was visualized via Cytoscape. The color intensity of the interspecies connective line was proportional to the correlation coefficient, where blue lines indicate inverse correlations and red lines indicate positive correlations.

In order to comprehensively identify and display the complex intra-boundary and inter-boundary relationships between the viruses and bacteria in the cattle diarrhea fecal samples, the Spearman correlation was used to test and calculate the correlation coefficient between the groups, and the relevant network was visualized using the Cytoscape software. The network association diagram analysis results show that there were complex intra-boundary and inter-boundary associations between the virome and bacterial community in the cattle diarrhea fecal samples. At the species level of virome, BoAstV interacted with bocaparvovirus 1, *Turicibacter*, *Shigella*, *Blautia*, *Butyricicoccus* (*P <* 0.05). BKV and nebovirus interacted synergistically (*P <* 0.05). Nebovirus interacted synergistically with *Pediococcu*, BKV, *Mogibacterium*, and calicivirus (*P <* 0.05). Rotavirus A and *Dorea*, Mamastrovirus 2, *Shigella*, *Erysipelotrichaceae*_*Clostridium*, *Lactococcus*, and *Agrobacterium* interacted with each other (*P <* 0.05). At the genus level of the bacterial community, *Shigella* interacted synergistically with BoAstV, rotavirus A, *Butyricicoccus*, and mamastrovirus 2 (*P <* 0.05). *Clostridiaceae_Clostridium* and coronavirus interacted synergistically (*P <* 0.05). *Clostridiaceae_Clostridium* interacted synergistically with *Psychrobacter* and *Collinsella* (*P <* 0.05). *Erysipelotrichaceae_Clostridium* interacted synergistically with *Oscillospira*, rotavirus A, and *Lactococcus* (*P <* 0.05). *Collinsella* and *Bifidobacterium*, *Oscillospira*, *Blautia*, *Subdoligranulum*, *Faecalibacterium*, *Olsenella*, *Clostridiaceae*_*Clostridium*, *Erysipelotrichaceae*_*Clostridium*, and *Dorea* interacted synergistically (*P <* 0.05), but antagonized with *SMB53* (*P <* 0.05). *Bifidobacterium* interacted synergistically with *Oscillospira*, *Faecalibacterium*, *Blautia*, *Ruminococcus*, and *Dorea* (*P <* 0.05). *Butyrivibrio* interacted synergistically with *SMB53*, BToV, Enterovirus (*P <* 0.05). *Butyricicoccus* interacted synergistically with BoAstV, *Faecalibacterium* (*P <* 0.05). *SMB53* and BKV interacted synergistically (*P <* 0.05). *SMB53* interacted synergistically with *Butyrivibrio*, *Facklamia*, and *Turicibacte* (*P <* 0.05), and antagonized with *Dorea* and *Collinsella* (*P <* 0.05) (Fig. 8).

### Epidemiological investigation and phylogenetic analysis of the 10 bovine enteric viruses identified in our study

**Fig 9:**
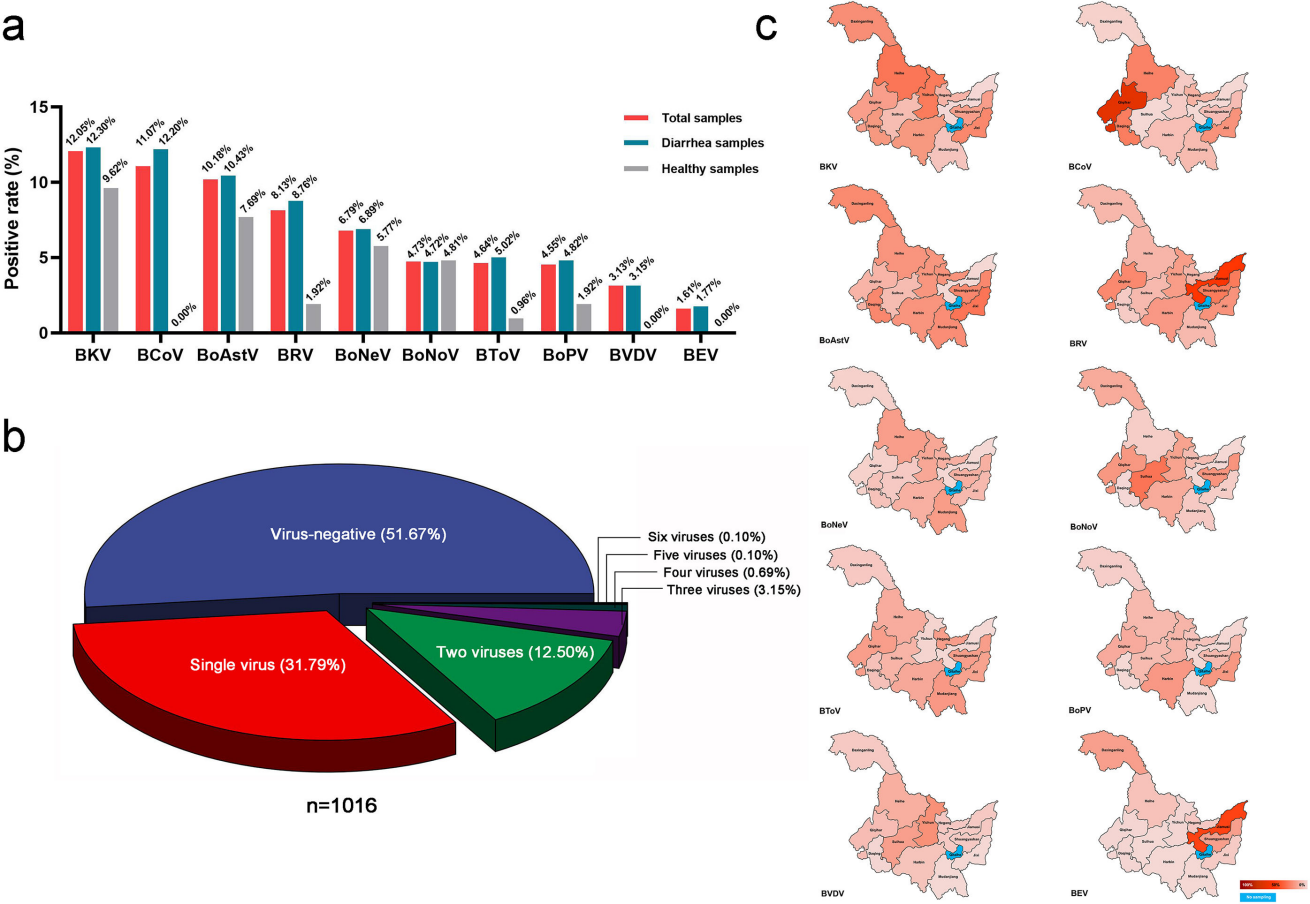
Positive rate and geographical distribution of 10 bovine enteric viruses. (**a**) Positive rate of 10 bovine enteric viruses. (**b**) Co-infection of 10 bovine enteric viruses. (**c**) Geographical distribution of 10 bovine enteric viruses.

In order to further investigate the infection of 10 bovine enteric viruses in Heilongjiang Province, a total of 1,120 samples were tested by RT-PCR for the pathogens of BKV, BToV, BoNoV, bovine nebovirus (BoNeV), bovine picornavirus (BoPV), BRV, BoAstV, BCoV, BEV, and BVDV. The results showed that the total positive rate of 10 bovine enteric viruses in 1,120 samples ranged from 1.61% to 12.05%, the positive rate of diarrhea fecal samples ranged from 1.77% to 12.30%, and the positive rate of healthy samples ranged from 0.00% to 9.62%. In 1,120 samples, the positive rates of 10 bovine enteric viruses BKV, BCoV, BoAstV, BRV, BoNeV, BoNoV, BToV, BoPV, BVDV, and BEV were 12.05% (135/1,120), 11.07% (124/1,120), 10.18% (114/1,120), 8.13% (91/1120), 6.79% (76/1,120), 4.73% (53/1,120), 4.64% (52/1,120), 4.55% (51/1,120), 3.13% (35/1,120), and 1.61% (18/1,120), respectively. The positive rate of 1,016 fecal samples from diarrhea showed that BKV was 12.30% (125/1,016), BCoV was 12.20% (124/1,016), BoAstV was 10.43% (106/1,016), BRV was 8.76% (89/1,016), BoNeV was 6.89% (70/1,016), BoNoV was 4.72% (48/1,016), BToV was 5.02% (51/1,016), BoPV was 4.82% (49/1,016), BVDV was 3.15% (32/1,016) and BEV was 1.77% (18/1,016). The positive rate of 104 healthy fecal samples showed that BKV was 9.62% (10/104), BCoV was 0.00% (0/104), BoAstV was 7.69% (8/104), BRV was 1.92% (2/104), BoNeV was 5.77% (6/104), BoNoV was 4.81% (5/104), BToV was 0.96% (1/104), BoPV was 1.92% (2/104), BVDV was 0.00% (0/104), and BEV was 0.00% (0/104) (Fig. 9a; Table S6). The analysis of co-infection detection results showed that, of the total 1,016 samples of diarrhea fecal, 48.33% (491/1,016) of the samples were positive for virus detection, while 16.54% (168/1,016) of the samples detected at least two viruses; of these, 12.50% (127/1,016) detected two viruses, 3.15% (32/1,016) detected three viruses, 0.69% (7/1,016) detected four viruses, and 0.10% (1/1,016) detected five and six viruses (Fig. 9b). The analysis of the detection results of cattle diarrhea fecal samples in different cities (regions) showed that the positive rate of BKV detection in cattle diarrhea fecal samples in Heilongjiang Province was the highest, and BToV, BoPV, and BoAstV were evenly distributed in all cities (regions) in Heilongjiang Province, with only small regional differences. Among the 12 cities (regions) in Heilongjiang Province, Jiamusi City has the smallest number of virus types detected in cattle diarrhea fecal samples, with only BRV and BEV detected (Fig. 9c; Table S7).

**Fig 10:**
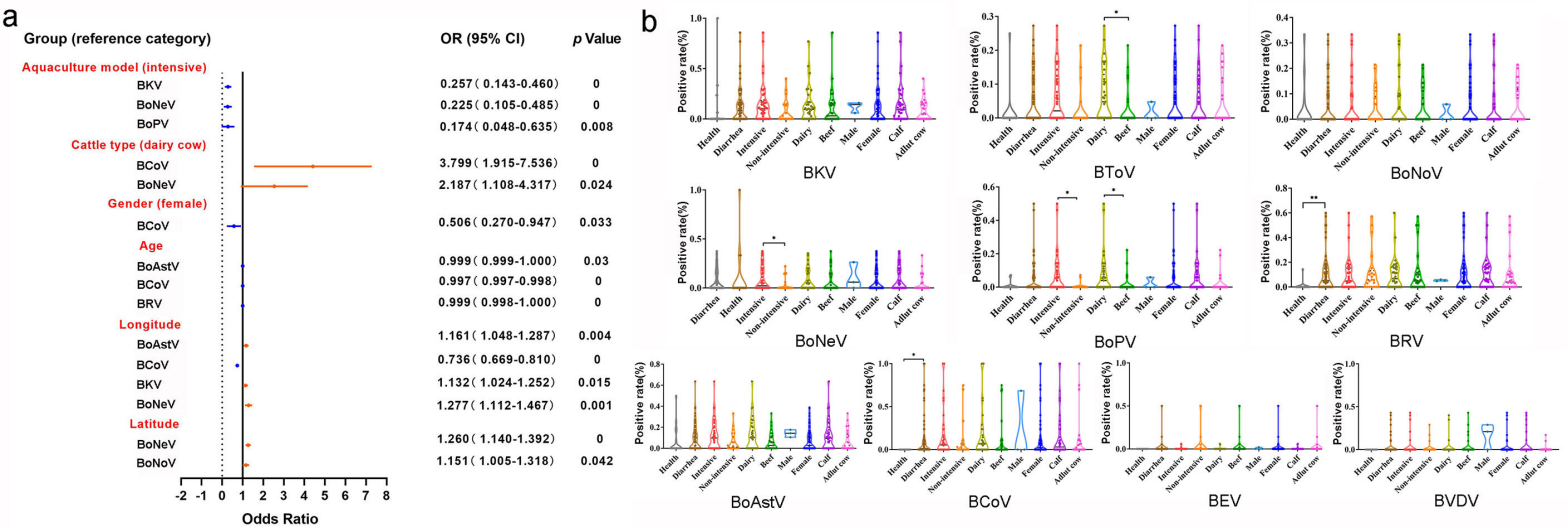
Correlation analysis between positive rate of 10 bovine enteric viruses and various factors. (**a**) Multivariate logistic regression analysis for different factors affecting 10 bovine enteric viruses. (**b**) Differential analysis of positive rate of 10 bovine enteric viruses under various factors.

In order to analyze the correlation between 10 bovine enteric viruses and different hosts (clinical status, type of cattle, gender of cattle, and age of cattle) and environmental factors (aquaculture model and geographical location), the binary logistic regression (Analyze-Regression-Binary Logistic) analysis, and SPSS statistical analysis (chi-squared test) were carried out. The results of Analyze-Regression-Binary Logistic showed that the infection of BKV, BoNeV, and BoPV was significantly correlated with the aquaculture model (*P <* 0.05); BCoV and BoNeV infections were significantly correlated with cattle type (*P <* 0.05); BCoV infection was significantly correlated with cattle gender (*P <* 0.05); BoAstV, BCoV, and BRV infections were significantly correlated with the age of cattle (*P <* 0.05); BoAstV, BCoV, BKV, and BoNeV infections were significantly correlated with longitude (*P <* 0.05); BoNeV and BoNoV infections were significantly correlated with latitude (*P <* 0.05) (Fig. 10a). SPSS statistical analysis (chi-squared test) showed that the infection of BRV and BCoV was significantly correlated with diarrhea (*P <* 0.05). The infection of BToV, BKV, BoNeV, BoPV, BoAstV, BCoV, and BEV was significantly correlated with the aquaculture model (*P <* 0.05). The infection of BToV, BoPV, BCoV, and BEV was significantly correlated with the type of cattle (*P <* 0.05) (Table 2). The positive rate of BRV and BCoV in the fecal samples of bovines with diarrhea was significantly higher than that in the healthy group (*P <* 0.05). The positive rates of BToV and BoPV in dairy cow diarrhea fecal samples were significantly higher than those in beef cattle (*P <* 0.05), and the positive rates of BoNeV and BoPV in intensively farmed cattle diarrhea fecal samples were significantly higher than those in non-intensively farmed cattle (*P <* 0.05) (Fig. 10b). This result was in line with the identification of virome in our study.

**TABLE 2:** Correlation analysis between different factors and 10 bovine enteric viruses identified in this study

Virues	Clinical status (diarrhea)	Aquaculture model (D-intensive)	Cattle type (D-dairy cow)
BKV	0.423	**0.000**	0.426
BToV	0.103	**0.026**	**0.004**
BoNoV	1.000	0.304	0.907
BoNeV	0.665	**0.002**	0.455
BoPV	0.270	**0.000**	**0.000**
BRV	**0.015**	0.467	0.278
BoAstV	0.379	**0.014**	**0.016**
BCoV	**0.000**	**0.006**	0.052
BEV	0.271	**0.001**	**0.001**
BVDV	0.127	0.183	0.240

^*a*^ Differences in values were considered statistically significant or highly significant at *P* < 0.05 or *P* < 0.01, respectively. Statistically significant or highly significant in bold. D-intensive is diarrhea cattle with intensive farming, D-dairy cow is diarrhea dairy cow.

**Fig 11:**
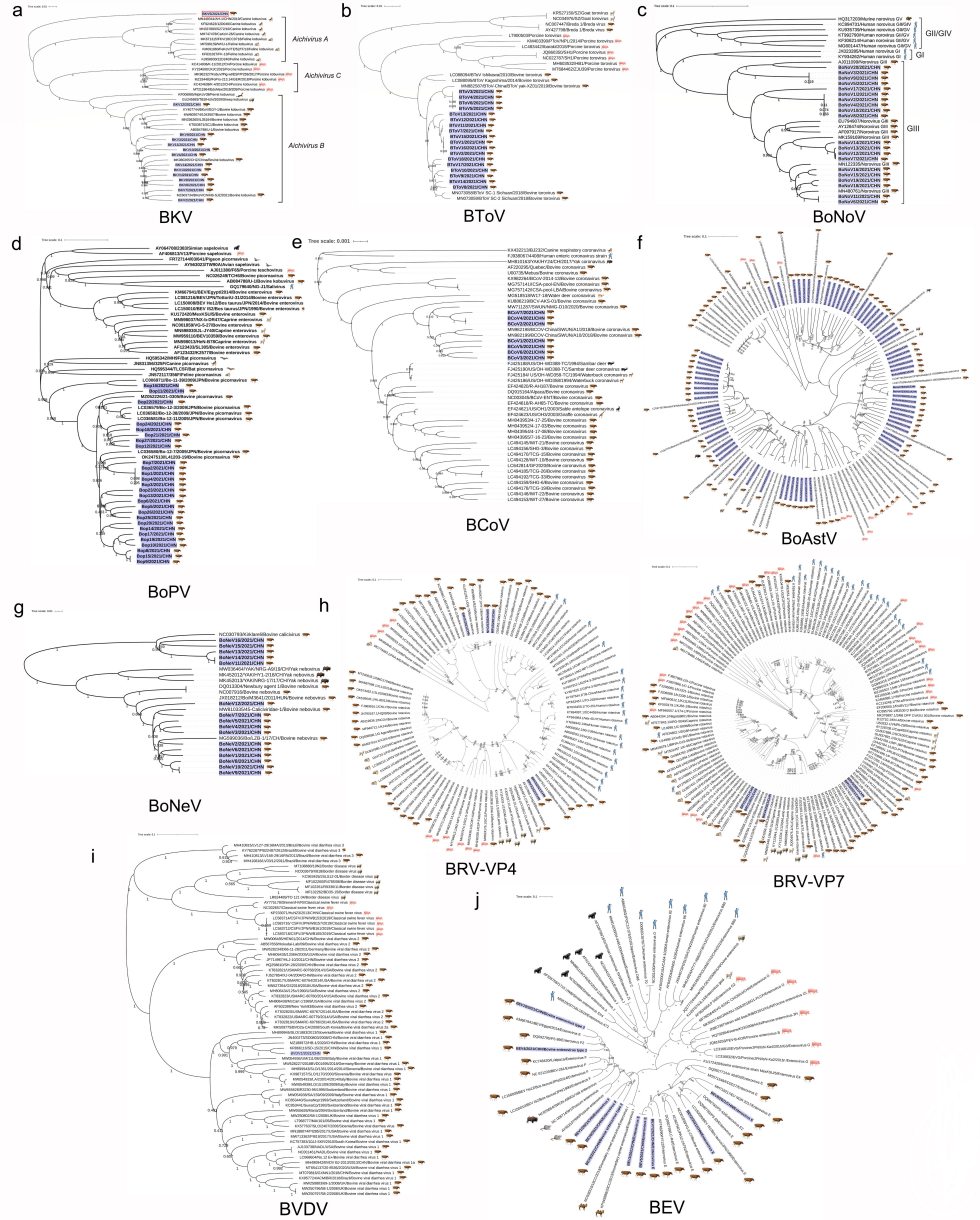
Phylogenetic analysis of 10 identified bovine enteric viruses.

In this study, 191 complete genome sequences of 10 bovine enteric viruses were identified. In order to determine the genetic evolution of the identified virus sequences, phylogenetic tree based on the whole-genome sequence was constructed. The results showed that all 10 bovine enteric viruses showed genetic diversity, and multiple viruses showed coevolution of multiple host animals, with potential for cross-species transmission. The phylogenetic analysis of BKV showed that, except for BKV5/2021/CHN, which was divided into a small branch, all identified BKV sequences, as well as the sheep kobuvirus (GenBank accession number: GU245693) and the ferret kobuvirus (GenBank accession number: KF006985), aggregated to form the aichivirus B group (Fig. 11a). In order to further clarify the genetic evolution of BKV5/2021/CHN, sequence alignment, and Bayesian analysis were performed on the identified BKV strains. The BKV genome identified in this study and the deduced amino acid (aa) sequence were compared with the sequences of seven representative kobuvirus species previously reported. The results showed that the nucleotide sequence of BKV5/2021/CHN gene was the most similar to Kagoshima-2-24-KoV of aichivirus D group, with 81.1% homology and 50.7–66.7% homology with other kobuvirus sequences. BKV5/2021/CHN strain and aichivirus D2 strain 2–24 KoV have the highest amino acid homology (85.7%), and the homology of different proteins was 69.0% (P1), 97.9% (2C), and 96.1% (3CD), respectively (Table S8). The amino acid sequence alignment of 14 BKV strains identified in this study showed that the three key amino acid patterns KDELR, YGDD, and FLKR located in the 3D region were highly conservative. The amino acid motif of HWAL located in 2A region was mutated into HWAI, and NCTHFV was mutated into NCTTFV. Point mutations were also found in the nucleic acid binding region of the picornavirus helicase at positions 131–138 in the 2C region (Fig. S1a). Next, the Bayesian phylogenetic tree based on BKV5/2021/CHN VP1 sequence was reconstructed. The results showed that the genetic relationship between BKV5/2021/CHN and Kagoshima-2-24-KoV/2015/JPN (GenBank accession number: LC055960) was the closest in this study, and it was aggregated with Kagoshima-1-22-KoV/2014/JPN (GenBank accession number: LC055961) to form aichivirus D group (Fig. S1b). Phylogenetic analysis of BToV strains showed that different species of torovirus aggregated into different branches. This study identified that BToV strains were divided into three different branches (Fig. 11b). According to the phylogenetic analysis of BoNoV, the BoNoV identified in this study belonged to GIII type. BoNoV20/2021/CHN has the closest relationship with the Jena strain (GenBank accession number: AJ011099). BoNoV17/2021/CHN was separately aggregated into a branch. Other BoNoV strains identified in this study aggregated to form three different branches (Fig. 11c). In addition, the phylogenetic analysis of BoPV showed that BoPV16/2021/CHN and Bo-11-39/2009/JPN strains (GenBank accession number: LC006971) aggregated to form a branch. BoPV11/2021/CHN strain formed a branch independently. Other BoPV strains identified in this study gathered to form a large branch (Fig. 11d). The BCoV identified in this study was divided into two branches, of which BCoV2/2021/CHN, BCoV4/2021/CHN, BCoV7/2021/CHN strains, and SWUM/NMG-D10/2020 (GenBank accession number: MW711287) converged to form one. BCoV1/2021/CHN, BCoV3/2021/CHN, BCoV5/2021/CHN, and BCoV6/2021/CHN strains had close genetic relationship with BCoV China/SWUN/A1/2018 (GenBank accession number: MN982198) and BCoV-China/SWUN/A10/2018 (GenBank accession number: MN982199), and gathered to form another branch (Fig. 11e). The phylogenetic analysis of BoAstV showed that the strains identified in this study could be divided into five branches. Among them, the BoAstV47/2021/CHN strain was closely related to the BoAstV/JPN/Hokkaido12-25/2009 (GenBank accession number: LC047793), and was closely related to the BoAstV2/2021/CHN strain, the porcine astrovirus AstV5-US-1A122 (GenBank accession number: JX556693), and PAstVB5-AH29-2014 (GenBank accession number: MT642595), forming a branch that was far from the genetic relationship with other identified BoAstV strains (Fig. 11f). The phylogenetic analysis of BoNeV showed that the identified BoNeV strains gathered to form three branches (Fig. 11g). Based on the VP4 and VP7 genes of the BRV strain, a phylogenetic tree was constructed to analyze its genetic evolution. In phylogenetic tree based on VP4 gene, BRV1/2021/CHN, BRV2/2021/CHN, and BRV4/2021/CHN strains were clustered in the same branch. BRV1/2021/CHN was the closest to China’s DQ-75 strain (GenBank accession number: GU181281) and Australia’s B-11 (GenBank accession number: AY047488). The phylogenetic tree analysis of the BRV VP7 gene was similar to that of the BRV VP4 gene. BRV1/2021/CHN, BRV2/2021/CHN, and BRV4/2021/CHN strains were closely related to HM26 strain (GenBank accession number: FJ545658), XJX-07 strain (GenBank accession number: EU828784), and UCD/RVL-Bov 2 strain (GenBank accession number: GQ433985) were clustered in the same branch (Fig. 11h). Only one full-length sequence of BVDV genome was obtained in this study. The BVDV1/2021/CHN identified in this study has the closest genetic relationship with the Chinese strain SD-15 (GenBank accession number: KR866116) (Fig. 11i). The BEV strains identified in this study were divided into two branches: BEV2/2021/CHN, BEV3/2021/CHN, BEV5/2021/CHN, BEV7/2021/CHN, BEV8/2021/CHN, and EV NGR 2017 strains (GenBank accession number: MH719217), BEV IS1/Bos taurus/JPN/1990 strains (GenBank accession number: LC150009), and HY12 strains (GenBank accession number: KF748290), which were closely related and formed a branch. BEV1/2021/CHN, BEV4/2021/CHN, BEV6/2021/CHN, BEV9/2021/CHN, BEV10/2021/CHN strains, and possum enterovirus W1 strain (GenBank accession number: NC008714) and dromedary enterovirus 20 CC strain (GenBank accession number: KP345888) gathered to form another large branch (Fig. 11j).

### Phylogenetic analysis of other eukaryotic viruses

**Fig 12:**
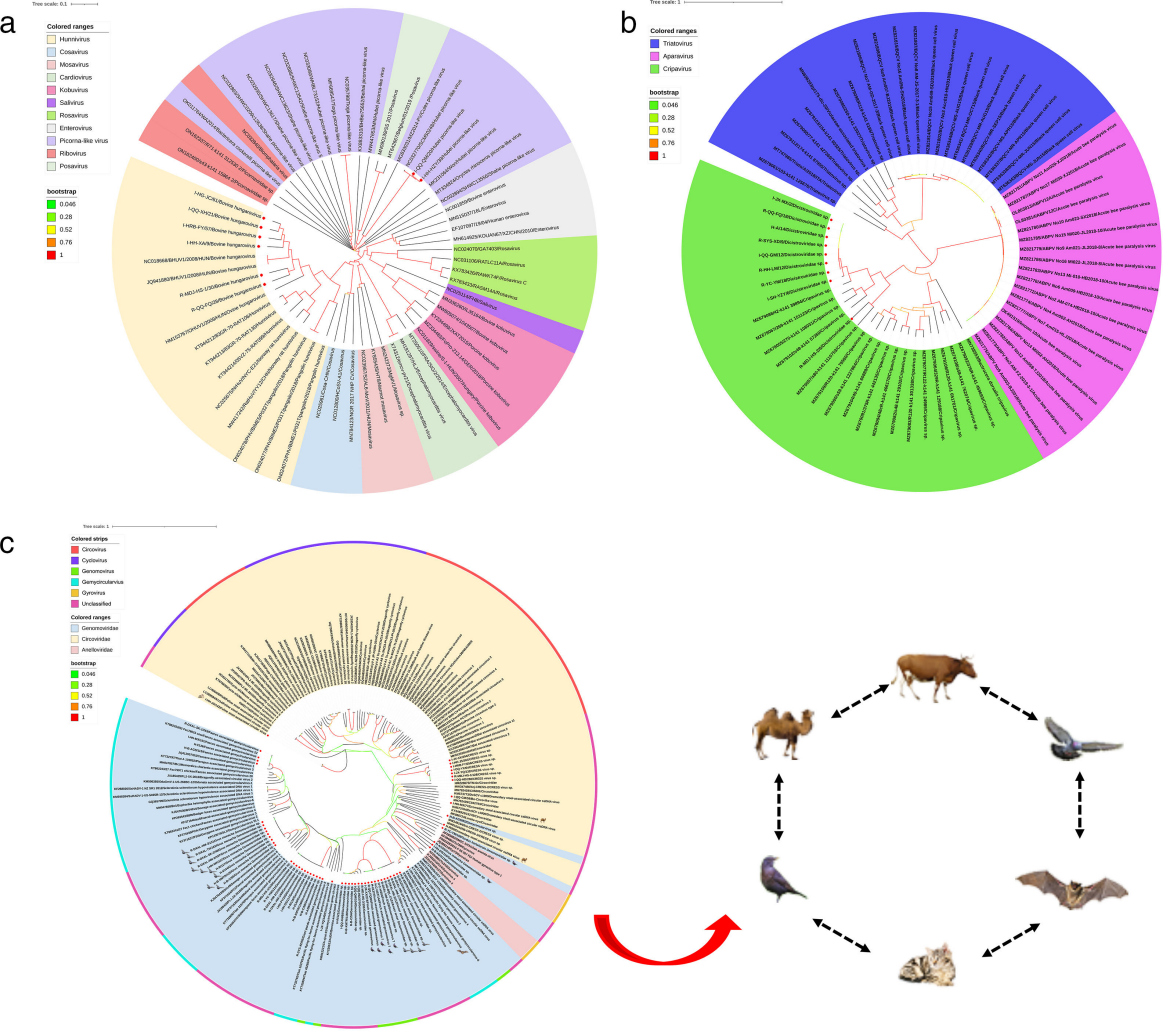
Phylogenetic analysis of other eukaryotic viruses. (**a**) Phylogenetic analysis based on complete genome of *Picornaviridae* strains. (**b**) Phylogenetic analysis based on complete genome of *Dicistroviriade* strains. (**c**) Phylogenetic analysis based on Rep protein of CRESS-DNA virus strains.

In the current study, 79 other eukaryotic virus genomes were found in dairy cow fecal samples, including 8 *Picornaviridae*, 9 *Dicistroviriade*, 51 *Genomoviridae*, and 11 *Circoviridae*. Phylogenetic analysis of *Picornaviridae* showed that six of the sequences belonged to bovine hungarovirus and two belonged to picorna-like virus (Fig. 12a). Among them, six Hungarian viruses came from cattle fecal samples from Hegang City, Qiqihar City, Harbin City, Heihe City, and Mudanjiang City. Two picorna-like viruses came from cattle fecal samples in Qiqihar and Heihe. The *Picornavirida* strains identified in this study belong to a branch together with viruses from other hosts. Phylogenetic analysis of *Dicistroviriade* showed that nine *Dicistroviriade* viruses belong to a cluster, namely, the Cripavirus group (Fig. 12b). They were from cattle fecal samples in Jixi City, Qiqihar City, Shuangyashan City, Heihe City, Yichun City, Suihua City, and Mudanjiang City, and one of them was from health sample. CRESS-DNA genome encodes replication-related proteins (Reps), mainly including *Circoviridae*, *Genomoviridae*, *Smacoviridae*, *Geminiviridae*, *Nanoviridae*, and *Bacilladnaviridae*, but many members had not been classified. They widely exist in various samples (lakes, sewage or soil, etc.), plant samples, and terrestrial animal samples. The phylogeny of CRESS-DNA viruses was divided into two main branches. The sequence of CRESS-DNA virus identified in cattle fecal samples in this study was close to the genetic relationship of other species and had the potential for cross-species transmission (Fig. 12c).

## DISCUSSION

In comparison to the strictly enclosed intensive farming modality employed for livestock like poultry and pigs, cattle farming involves both an intensive farming modality and a familial farming modality. The ranch was usually in a relatively open environment. Cattle were frequently exposed to human activities and environmental factors, and the pathogenic biological factors causing bovine diarrhea disease were also more complex. The infection of pathogenic microorganisms and the change of intestinal flora and virome were the most important factors leading to the occurrence of bovine diarrhea. Previous research had mainly concentrated on the investigation of diarrhoea-causing microorganisms in the context of single disease-related ecological factors (8, 15, 18). The research population was generally limited in size, and there was an absence of systematic disease ecological factors such as pathogen-host-environment. Due to the deficiency of microbial information concerning bovine diarrhea, the comprehensive risk factors of its occurrence have yet to be determined. Such as the type of pathogenic microorganism, the interaction between pathogenic microorganism and host factor, the interaction between pathogenic microorganism and environmental factor, and the interaction between host and environmental factor are yet to be fully understood. Despite ongoing research, the cause of bovine diarrhea remains unknown and existing strategies for its prevention and control are not as effective as hoped (8). For this study, the cattle herd in Heilongjiang Province was chosen as the research object. In their natural environment, fecal samples were collected from both cattle with diarrheic and healthy cattle. By considering the information from virology and bacteriology, a thorough evaluation of the major ecological risk factors of bovine diarrhea disease was conducted to uncover the features of virus and bacterial community present in the fecal samples of diarrheic and healthy cattle in various disease ecological backgrounds. Investigating the correlation between the composition and abundance of virome and bacterial community and host and environmental factors, as well as the prevalence, genetic evolution, cross-species transmission, and pathogenicity of common bovine enteroviruses in cattle in relation to host and environmental factors was essential in order to gain a better understanding of the laws that govern them. For the first time, a large sample volume of virology and bacteriology studies has revealed the disease risk factors related to the occurrence of bovine diarrhea disease, providing a new concept for the formulation of efficient comprehensive prevention and control measures for bovine diarrhea disease.

The term “microbiome” was first used as a convenient ecological framework for testing biological control systems, namely, the microbiome. This can be defined as a characteristic microbial community occupying a reasonably defined habitat that has unique physiological and chemical properties (19). At present, research on the human intestinal microbiota has proved that the microbiota participates in a series of physiological processes that are critical to the host’s health. The report showed that changes in the gastrointestinal microbiota were not only significantly related to human inflammatory intestinal diseases, but also to asthma, obesity, metabolic syndrome, cardiovascular diseases, immune-mediated diseases, and neurodevelopmental diseases (19, 20). Among the reported studies on the bovine microbiome, it was mainly focused on rumen microorganisms, including rumen microorganisms and feed digestion, changes in the rumen microbial composition with the growth of cattle age, and rumen microorganisms and methane emissions (21–23). In a few studies on the bacteriology of cattle dung, *Akkermansia*, *Solibacillus*, *Escherichia-Shigella*, *Alistipes*, *Solibacillus*, *Bacteroides*, *Prevotelaceae*, and *Bacillus* were significantly enriched in the diarrhea samples (12, 13). This study identified 39 bacterial phyla and 1,011 bacterial genera in fecal samples. In addition to the pathogenic bacteria identified in the previous studies, *Enterococcaceae_Enterococcus* and *Clostridiaceae_Clostridium* were identified in diarrhea fecal samples under different risk factors, and the important correlation of probiotics in health and diarrhea fecal samples was updated. Viromics research of other animals was mainly concerned with the identification of novel viruses (24) and the investigation of the cause of diseases (25). In the viromics study of other organ systems in cattle, the potential pathogenicity of influenza D virus (IDV) in the respiratory tract was determined through virus metagenomics identification (26). The BoAstV (BoAstV-CH13/NeoS1) and a new bovine double retrovirus, BoRV-CH15, were found in the samples of non-suppurative encephalitis in cattle. Five new CRESS-DNA viruses encoding replication-related proteins and three bovine parvoviruses have been identified in bovine plasma (26). In the macrogenomics sequencing study of cattle fecal samples, 26 viral families were identified and it was found that the reads of calicivirus and astrovirus in diarrhea calves were much higher than those in healthy calves (15, 18). In this study, 39 virus families, 86 virus genera, and 110 virus species were identified in the fecal samples of cattle, which greatly enriched the virology study of cattle diarrhea fecal. In short, compared with other studies, the sample size in this study was large, and the types of bacteria and viruses identified were more complex. Not only that, but most of the other microbiological studies have only carried out viromics or bacteriomics studies, respectively. This was the first time that viromics had been combined with bacteriomics in fecal samples from cattle with diarrhea, comprehensively revealing and expanding the role of virology and bacteriology in diarrhea. The relationship between the virome at the family level and the bacterial community at the genus level and different factors of diarrhea and health was identified by making full use of the information of the bacterial community and the virome. The relationship between the changes of bovine gastrointestinal microflora and bovine diarrhea disease under different factors has been clarified in terms of bacteria and viruses.

Host microbiome plays an important role in host immunity, is at the interface between the host and pathogen, and affects disease outcomes. In recent years, accumulated research has shown that the intestinal microbiota is related to many diseases (27–29). Environmental and host factors affect the diversity of the intestinal flora. In many cases, the interaction between the host, the pathogen, and the environment was not sufficient to describe the disease dynamics in the field. The interaction between the host, a host microbiome, a pathogen, and the environment affects the occurrence of disease. In order to fully reveal the pathogenesis of diseases, it is necessary to transform the disease triangular model of host, pathogen, and environment into a disease tetrahedral model of host, host microbiome, pathogen, and environment. Bernardo-Cravo et al. (30) reported that environmental factors and the host microbiota affect the host-pathogen dynamics (30). By integrating environmental factors and microbiota into the pathogenesis of disease, we can improve our understanding of the disease dynamics and ecology (31). In our study, we have deeply analyzed the correlation between the virome and bacterial community and the host and environmental factors. The composition, abundance, and diversity of the viral and bacterial community in the diarrhea of cattle feces show different degrees of changes under different host and environmental factors. In cattle with diarrhea, the abundance of 16 virus species and 17 bacterial genera was significantly correlated with host factors (clinical status, type of cattle, and age of cattle) or environmental factors (aquaculture model and geographical location) (*P <* 0.05). In this study, under intensive farming conditions, the abundance of *Coronaviridae* and *Astroviridae* in the diarrhea of cattle fecal samples showed a high abundance, and the abundance of *Piconaviridae*, *Coronaviridae*, and *Reoviridae* in cattle diarrhea fecal samples also showed a high abundance. The correlation analysis between the identified virome and bacterial community and the environment or host indicates that the abundance of *Picornaviridae*, *Coronaviridae*, and *Caliciviridae* was significantly correlated with diarrhea (*P <* 0.05). BCoV in *Coronaviridae* has been proved to cause severe calf diarrhea. The *Picornaviridae* and *Caliciviridae* have been reported to be frequently detected in the diarrhea fecal samples in previous studies, but it has not been confirmed that they can independently cause diarrhea. In addition, this study showed that some potential diarrheic pathogens were significantly correlated with different environmental factors in the fecal samples of diarrhea; for example, the abundance of *Astroviridae* was significantly correlated with an intensive farming mode (*P <* 0.05), and the abundance of *Circoviridae* was significantly correlated with the non-intensive farming mode (*P <* 0.05). At the same time, some viruses were significantly correlated with host factors, and the abundance of *Astroviridae* and *Reoviridae* was significantly correlated with the calves (*P <* 0.05). Our research verified that various viruses in the real environment can cause diarrhea, a consequence that was influenced by both host and environmental factors. Additionally, this study revealed differences in the abundance of diarrhea-causing bacteria in diarrheic fecal samples under an aquaculture model. In intensive farming and dairy cow, there were many pathogenic bacteria in diarrheic fecal samples, such as *Shigella* and *Enterococcaceae_Enterococcus* has a high abundance and belongs to the dominant bacteria genus. However, the difference in the performance of diarrhea fecal samples in the non-intensive farming mode suggests that the pathogenic bacteria of bovine diarrhea were more likely to gather in the intensive farming or dairy cow, and that the pathogenic bacteria of diarrhea may cause more serious harm to the intensive farming dairy cow. It was evident from the data that the virus and bacteria associated with bovine diarrhea have an association with host factors such as clinical status, type of cattle, and age of cattle, as well as environmental factors such as an aquaculture model and geographical location. This study has yielded new information regarding the prevalence of bovine diarrhea disease and has provided us with advice on how to curb and combat the bovine diarrhea virus. In summary, the chi-squared test analysis display of this study revealed 10 viral families, including *Virgaviridae*, *Totiviridae*, *Siphoviridae*, *Reoviridae*, *Picornaviridae*, *Herpesviridae*, *Genomoviridae*, *Coronaviridae*, *Caliciviridae*, *Astroviridae* as well as the abundance of 17 bacterial genera of *Mogibacterium*, *SMB53*, *Erysipelotrichaceae_Clostridium*, *Acidovorax*, *Actinomyces*, *Lactococcus*, *Collinsella*, *Staphylococcaceae_Staphylococcus*, *Olsenella*, *Aerococcus*, *Butyricicoccus*, *Chryseobacterium*, *Subdoligranulum*, *Carnobacterium*, *Trichococcus*, *Faecalibacterium*, and *Planomicrobium* was significantly correlated with different host factors (clinical status and type of cattle) and environmental factors (aquaculture model) (*P <* 0.05), and the interaction network diagram appears, providing a new way of thinking about the occurrence of diarrhea disease. In contrast to past research, we have extensively examined the interplay between virome and bacterial community, distinct environments, and hosts, thereby augmenting our comprehension of the interaction between host, host microbial group, pathogen, and environment when bovine diarrhea disease occurs.

Disease is a complicated phenomenon that results from the interaction between a pathogen and the host. Research on human and animal diseases has verified that a synergistic pathogenic effect exists between bacteria and bacteria, viruses and viruses, and bacteria and viruses during the emergence of a disease (32, 33). The complexity of the occurrence of diarrheic diseases was heightened by the interaction and synergism between intestinal microorganisms, and understanding this mechanism can help in the prevention and control of such diseases. In our research, we conducted a thorough investigation into the interplay between the discovered pathogens. A notable synergy exists between 12 pairs of viruses, 96 pairs of bacteria, and 22 pairs of bacteria and viruses (*P <* 0.05). In terms of the interaction between bacteria and bacteria, in fecal samples of diarrhea, *Shigella*, and *Enterococcaceae_Enterococcus* interact with each other (*P <* 0.05). In different aquaculture models, *Clostridiaceae_Clostridium* and *Streptococcus* interact with each other in the fecal samples of intensive breeding cattle diarrhea (*P* < 0.05). *Shigella*, *Enterocacaee_Enterococcus*, and *Streptococcus* interact synergistically (*P* < 0.05). Furthermore, *Shigella* interacted with *Streptococcus* in the fecal samples of non-intensive cattle diarrhea (*P* < 0.05). Among different types of cattle, in dairy cow diarrhea fecal samples, *Enterococcaceae_Enterococcus* and *Shigella* interacted synergistically (*P* < 0.05). Previous studies have reported that Porcine circovirus type 2 (PCV2) can promote the infection of other DNA viruses by inhibiting the induction of type I interferon (32), suggesting that the virus can interact with other viruses or promote the pathogenesis. It had also been reported that, when BoAstV was mixed with BRV or BToV infection, calves had more serious diarrhea in bovine enterovirus (34). In this study, a variety of potential bovine diarrhea viruses, including BKV, BToV, BoAstV, nebovirus, and calicivirus, were significantly co-pathogenic. Enterovirus and BToV, BoAstV BKV, and bocaparvovirus 1 were significantly co-pathogenic. In addition, this study found that there was a complex inter-boundary relationship between the viral and bacterial boundaries in the diarrhea cattle fecal samples. Previous studies have found that the interaction between bacteria and viruses can play a key role in the host-pathogen interface (35). Co-infection of a virus and bacteria may be recognized as a risk factor for the development of severe secondary diseases (36). Several reports have demonstrated the existence of direct or indirect interactions between different intestinal pathogenic viruses and bacteria, thereby influencing their respective pathogenicity. For example, the direct binding of bacterial surface polysaccharides can enhance the stability of enterovirus particles and increase their adhesion to host receptors (33), the interaction between reovirus and bacteria enhances the thermal stability of the viruses (37), and infection by CagA-positive *Helicobacter pylori* induces expression of GII.4 norovirus attachment factors in nonsecretors' mucosa, expanding the host range of these strains (38). These strategies emphasize the cooperation mechanism between viruses and bacteria to realize mutual benefits through cross-generational interaction. In this study, multiple pathogenic bacteria causing diarrhea and potential viruses causing diarrhea showed synergistic pathogenic effects, such as *Shigella* and BoAstV, rotavirus A. *Erysipelotrichaceae_Clostridium* interacted with rotavirus A. *Clostridiaceae_Clostridium* interacted with coronavirus. The above research results well explain why the isolated pathogen does not occur in the infection experiment, and why we have not identified the “recognized pathogen” in some serious diarrhea diseases. The relevant data promote our further understanding of the interaction mechanism between pathogenic microorganisms in the occurrence of bovine diarrhea.

In recent years, host intestinal factors, especially intestinal flora, have become a hot spot (39, 40). In this study, under the background of comprehensive disease ecology, we found that the abundance of *SMB53*, *Butyrivibrio*, *Facklamia*, *Trichococcus*, *and Turicibacter* was significantly correlated with health (*P <* 0.05), with a trend of antagonizing diarrhea. Moreover, some probiotics and other pathogenic bacteria and viruses show complex interactions, such as the interaction between *SMB53* and BKV. *Butyrivibrio* interacted with BToV and enterovirus. *Turicibacter* and *Shigella*, *Enterococcaceae_Enterococcus*, and *Clostridiaceae_Clostridium* interaction. Furthermore, different aquaculture models have varying interactions between pathogens and probiotics. *Clostridiaceae_Clostridium* interacted with *Blautia* and *Butyricicoccus*. In the case of non-intensive aquaculture model or beef cattle diarrhea fecal samples, *Clostridiaceae_Clostridium* interacted with *Turicibacter* and *Bifidobacterium*. This study found the synergy or antagonism between a variety of probiotics and bacteria and viruses, and the interaction was different under different environmental factors. Previous reports have shown that the intake of probiotics was related to better control of infectious diseases, and in some cases, the duration or severity of the infection can be improved (41, 42). In view of the complex interaction between probiotics and intestinal bacteria in this study, we speculate that the interaction between probiotics and intestinal bacteria was also an important mechanism that affects the development of disease. In addition, probiotics, such as *Collinsella* and *Bifidobaterium*, have high abundance in many healthy fecal samples. In the beef cattle diarrhea fecal samples, *Collinsella* and *Clostridiaceae_Clostridium* also have antagonistic effects. At present, it is known that *Collinsella* is a probiotic in the intestinal tract that has the effect of inhibiting inflammation, inhibiting cell apoptosis, and anti-oxidation in the intestinal tract. The latest research has confirmed that *Collinsella* even plays a role in the inhibition of COVID-19, which may cause intestinal symptoms (43). *Bifidobacterium* also has many prebiotic functions in the human intestine, such as improving intestinal diseases caused by immune system disorders and inhibiting the invasion of pathogenic bacteria into the intestine (44). A variety of probiotics identified in this study were significantly related to health, and some of these probiotics exist in high abundance in healthy samples. These probiotics may be essential for sustaining the well-being of the bovine intestines and counteracting bovine diarrhea. Additionally, they could be used to create antiviral treatments in the future, which would be highly beneficial for the prevention and management of diarrhea diseases in cattle and healthcare.

Owing to the complexity of the bovine intestinal environment, there was a divergence of views among researchers regarding the pathogenicity of intestinal pathogens. At present, it has been confirmed that BRV, BCoV, BVDV, and so on, can cause calf diarrhea. Other viruses that have been reported to may cause diarrhea include BoAstV, BToV, BoPV, BoNeV, and so on (9, 45). Researchers have revealed that a variety of related viruses can be found in bovine diarrhea, yet the prevalence of bovine enteric viruses in masses remains uncertain (46–48). In our study, 10 kinds of bovine diarrhea-associated viruses, including BKV, BToV, BoNoV, BoNeV, BoPV, BRV, BoAstV, BCoV, BEV, and BVDV, were found in virome. A large-scale retrospective epidemiological survey was conducted for 10 kinds of bovine enteric viruses. The results showed that the total positive rate of the 10 kinds of bovine enteric viruses was 1.61–12.05%, and the positive rate of diarrhea fecal samples was 1.77–12.30%. Among them, the positive rates of diarrhea fecal samples of BRV, BCoV, and BVDV were 8.76% (89/1016), 12.20% (124/1016), and 3.15% (32/1016), respectively. Compared with previous research, the infection rate of BRV was slightly lower. This may be due to the large sample size of this study, and the fact that the sample collection includes adult cattle. However, in the further statistical analysis of infection rate, we found that the positive rate of BRV and BCoV detection in diarrhea fecal samples was significantly higher than that in healthy samples, and the analysis showed that the infection of BRV and BCoV was significantly correlated with diarrhea (*P <* 0.05), which was consistent with the previously reported clinical results that BRV and BCoV were the main pathogens causing bovine diarrhea. In addition, BRV was significantly correlated with the age of cattle (*P <* 0.05), and BCoV infection was significantly correlated with the aquaculture model (*P <* 0.05). The retrospective testing’s statistical analysis outcomes exhibit conformity with the outcomes of metagenomic identification. The positive rate of BKV, BToV, BoNoV, BoPV, BoAstV, BoNeV, and BEV among other viruses that may cause enteritis ranges from 1.77% (18/1016) to 12.30% (125/1016), with BKV having the highest infection rate of 12.30% (125/1016). In addition, in the total 1,016 fecal samples of diarrhea, the positive rate of virus detection accounts for 48.33% (491/1016), while 16.54% (168/1016) samples detect at least two viruses, which proves that bovine viral diarrhea was widespread, and virus co-infection was common. BKV was also the main co-infection virus, which virome identification has confirmed interacted with a variety of viruses. Furthermore, BKV infection was found to be significantly related to the aquaculture model (*P* < 0.05). Results have demonstrated that BKV may be the most essential co-diarrhea virus in diarrhea fecal samples, and was a major factor in bovine diarrhea disease. Therefore, research related to BKV should not be overlooked. It was suggested that the environmental factors and microbial interaction should be taken into account when studying the pathogenicity of BKV. Additionally, BoAstV, a member of *Astroviridae*, has been gaining attention from researchers due to its potential for cross-species transmission (49). Numerous studies have shown that BoAstV is typically present in fecal samples from cattle with diarrhea, indicating a link between the virus and the disease (34, 49). However, the current research on BoAstV has not clarified its ability to cause diarrhea. In this study, the positive rate of BoAstV detection in diarrhea fecal samples was 10.43% (106/1,016), which was higher than that in healthy samples, at 7.69% (8/104). The results of SPSS statistical analysis showed that the infection of BoAstV was significantly correlated with the aquaculture model and age (*P <* 0.05). In addition, BoAstV and various other bovine enteric viruses showed synergistic pathogenicity in fecal samples from diarrheic cattle. These data indicate that the pathogenicity of bovine astrovirus may also be affected by interactions among different hosts, environments, and even other intestinal microorganisms in clinical settings. The infection of other viruses, such as BToV, BoNeV, BoPV, and BEV, was also significantly related to the aquaculture model and cattle type. At the same time, this study identified that most viruses were also affected by the complex interaction of other intestinal bacteria and co-infected viruses. In this study, there was an interaction between viruses and bacteria, and even between different environmental and host factors. Their interaction presents different changes. The interaction of these factors brings strong complexity to the occurrence of diarrhea. In conclusion, our research shows that viral diarrhea plays an important role in bovine diarrhea. We must strengthen the prevention and control of known and confirmed pathogenic BCoV and BRV, and pay high attention to the problem of mixed infection, as well as the formation of synergy between these pathogens. In addition, we should pay attention to the impact of the aquaculture model on the occurrence of diarrhea. The non-intensive aquaculture model may be an effective way to avoid cattle diarrhea. In the intensive aquaculture model, and when calf diarrhea occurs, we should pay attention to the isolation breeding, and try to avoid the widespread occurrence of diarrhea caused by the mutual transmission of viruses.

Cattle were ubiquitous, varied in breed, abundant in quantity, and closely connected to human life. Cattle breeding was largely exposed, with frequent contact between people and other animals, which has caused multiple bovine zoonosis cases and posed a great danger to biosafety. There were also some viruses in bovine enteric viruses, such as BoAstV, BCoV, and BRV, which have shown potential for cross-species transmission (45, 50, 51), and BCoV has even been reported to be isolated and identified in the fecal samples of children with diarrhea (52). Therefore, it was significant for biosafety to focus on cross-species, new virus mining, genetic evolution monitoring, the cattle farm biosafety prevention, and curbing the cross-species transmission of viruses at the front-end source. In this study, 191 complete genome sequences of 10 viruses in dairy cow fecal samples were identified through macro genome sequencing analysis of a large sample size, deep mining, and sequencing assembly, which greatly enriched the available complete virus genome database. Further, the genetic evolution of bovine diarrhea-associated viruses was comprehensively displayed through evolutionary analysis, and the phylogenetic analysis revealed the complexity and genetic diversity of the evolution of the important enteroviruses. The results showed that many kinds of viruses, such as BEV, BoAstV, and BoNoV, showed multiple branches of genetic evolution. BoAstV was also clustered with the buffalo astrovirus, the bovine respiratory astrovirus strain, and even the porcine astrovirus, which was of reference significance for analyzing the genetic evolution and even cross-species transmission of astroviruses. In addition, 70 other eukaryotic virus genomes were found in this study, of which 6 whole-genome sequences of the bovine hungarovirus 1 were identified. As far as we know, the bovine hungarovirus has only been detected in cattle in Hungary (53) and Turkey (54), and was found for the first time in dairy and beef cattle in China. In addition, CRESS-DNA viruses in the *Circoviridae* and *Genomoviridae* can infect a variety of eukaryotes, including mammals, birds, insects, fungi, and environmental samples. These viruses have high genetic diversity (55–59). The report shows that the infection of these viruses may play a role in the disruption of the host immune system and become an indirect factor in the causation of other diseases (60). Based on phylogenetic analysis, 62 CRESS-DNA virus genome sequences were found in the feces of cattle. The CRESS-DNA virus identified in this study has a close genetic relationship with many different species of hosts, indicating that these viruses have the potential to spread across species. In general, cattle may be an important host of many viruses, providing opportunities for cross-species transmission of viruses.

In conclusion, this study disclosed the composition of the virome and bacterial community in fecal samples of diarrheic cattle from Heilongjiang Province and their alterations under different host and environmental factors. There is a significant correlation between the prevalence of viruses and bacteria in cattle afflicted with diarrhea and various factors related to either the host (such as clinical status, age, and type of cattle) or the environment (such as the aquaculture model and geographical location). The coexistence of viruses-viruses, bacteria-bacteria, and bacteria-viruses in cattle with diarrhea leads to a significant synergistic effect. The 10 enteric viruses found in bovines exhibit a high incidence of infection, genetic variability, and coevolution across species in cattle with diarrhea. These viruses are linked to ecological factors that contribute to the development of disease, including the host and the environment. This study enriches the database of the bovine virome and bacterial community, and provides a new perspective for formulating comprehensive prevention and control measures of bovine diarrhea disease based on the ecological factors of the pathogen-host-environment system.

## MATERIALS AND METHODS

### Sample collection and preparation

A total of 1,120 fecal samples from 1,120 cattle belonging to 58 farms were collected in Heilongjiang Province, China, between June 2020 and November 2020 (Table 1; Table S1). These 1,120 cattle included 1,016 diarrheic cattle and 104 healthy cattle. 1,016 diarrheic cattle came from 34 dairy farms and 24 beef farms, covering 12 cities (regions) in Heilongjiang Province in northeast China, including Qiqihar, Daqing, Heihe, Jixi, Mudanjiang, Hegang, Harbin, Shuangyashan, Daxinganling, Yichun, Jiamusi, and Suihua. 104 healthy cattle come from 4 dairy farms and 14 beef farms, covering nine cities (regions), including Hegang, Heihe, Daqing, Harbin, Daxinganling, Mudanjiang, Jixi, Yichun, and Jiamusi. The diarrheic cattle were diagnosed by licensed veterinarians. The diarrheic cattle showed clinical manifestations of acute diarrhea. Gloved fingers were used to collect fecal samples from the rectum of each cattle, and gloves were replaced between each cattle. Separate fecal samples were placed in sterile bags and immediately stored on ice for transport to the laboratory, then stored at −80°C until further processing.

One thousand one hundred twenty samples were mixed into 72 sequencing samples, including 62 diarrhea fecal mixed samples (including 1,016 diarrhea fecal samples) and 10 healthy fecal mixed samples (including 104 healthy fecal samples). According to the disease ecological factors, 72 sequencing samples include different host factors: clinical status (diarrhea, health), cattle type (dairy cow: Holstein, beef cattle: Angus and Simmental), gender (male, female), cattle age (calves, adults), and environmental factors: aquaculture model (intensive, non-intensive), geographic location (eastern Heilongjiang, western Heilongjiang, southern Heilongjiang, northern Heilongjiang). The ecological background information of the diseases of the samples is shown in Table 1; Table S1.

### Viral metagenomic sequencing

To achieve equivalent input amounts of raw feces, the total composite sample weight was targeted at 1 g using an electronic balance. Fecal samples were re-suspended in two volumes of phosphate-buffered saline (PBS) and vigorously vortexed for 15 min. The fecal supernatants were collected after centrifugation for 10 min at 15,000 × *g*. About 500 µL of each supernatant was filtered through a 0.45-µm Millipore filter to remove large cell-sized particles. The filtrates enriched in viral particles were treated with DNase and RNase to digest unprotected nucleic acid at 37°C for 60 min. The remaining total nucleic acid, protected from digestion within viral capsids, was extracted using the TIANamp Virus RNA Kit and TIANamp Stool DNA Kit (Tiangen Biotech Co., Ltd., Beijing, China) according to the manufacturer’s instructions (61, 62). The viral nucleic acid samples were subjected to reverse transcription reactions using reverse transcriptase and random hexamers, and subsequent second-strand DNA synthesis. Out of 72 sequencing samples, 70 libraries were successfully constructed using Nextera XT reagents (Illumina) and sequenced on the NovaSeq 6000 (Illumina). The Illumina sequencing and library construction were performed at the Shanghai Tanpu Biotechnology Co., Ltd (Shanghai, China). Raw reads were filtered and trimmed by fastp (v0.20.0, https://github.com/OpenGene/fastp) to remove sequencing adapters and low-quality reads, including those reads scoredQ20 (Phred quality score of 20). Ribosomal RNAs and host reads subtraction by read-mapping were performed with BBMAP program (v38.51, https://sourceforge.net/projects/bbmap/files/). *De novo* genome assembly was performed using SPAdes v3.14.1 (63). These extracted assembled scaffolds limited the minimum contig length to 100 bases, with the best BLAST hits to NCBI nt database. In order to reduce the false positive mismatch of the reads caused by sequence homology, the mapped species with relative abundance higher than 1% could be conserved. The information of each viral library is shown in Table S2. To characterize the microbial communities, the heatmaps were generated using R (version 3.5.1, distance = “average,” scale = “row”) with the pheatmap package.

### Bacterial 16S rRNA sequencing

PCR amplification of the bacterial 16S rRNA genes V3-V4 region was performed using the forward primer 338F (5′-ACTCCTACGGGAGGCAGCA-3′) and the reverse primer 806R (5′-GGACTACHVGGGTWTCTAAT-3′). Sample-specific 7 bp barcodes were incorporated into the primers for multiplex sequencing. The PCR components contained 5 µL of buffer (5×), 0.25 µL of Fast pfu DNA Polymerase (5 U/µL), 2 µL (2.5 mM) of dNTPs, 1 µL (10 µM) of each Forward and Reverse primer, 1 µL of DNA Template, and 14.75 µL of ddH_2_O. Thermal cycling consisted of initial denaturation at 98°C for 5 min, followed by 25 cycles consisting of denaturation at 98°C for 30 s, annealing at 53°C for 30 s, and extension at 72°C for 45 s, with a final extension of 5 min at 72°C. PCR amplicons were purified with Vazyme VAHTSTM DNA Clean Beads (Vazyme, Nanjing, China) and quantified using the Quant-iT PicoGreen dsDNA Assay Kit (Invitrogen, Carlsbad, CA, USA). After the individual quantification step, amplicons were pooled in equal amounts, and pair-end 2 × 250 bp sequencing was performed using the Illumina MiSeq platform with MiSeq Reagent Kit v3 at Shanghai Personal Biotechnology Co., Ltd (Shanghai, China). Briefly, raw sequence data were demultiplexed using the demux plugin, followed by primers cutting with cutadapt plugin (64). Sequences were then quality filtered, denoised, merged, and chimera removed using the DADA2 plugin (65). The information of each bacterial library is shown in Table S4.

### Virome and bacterial community analysis of fecal samples from diarrhea-affected and healthy cattle with various environment and host conditions

Analyses were performed to compare the constituents of fecal microbiome among different factors. To characterize the microbial communities, the heatmaps were generated using R (version 3.5.1, distance = “average',” scale = “row”) with the pheatmap package. The analysis of virome and bacterial community composition varying with latitude and longitude was performed by GraphPad Prism 8.

### Correlations analysis of virome and bacterial community

The different factors (clinical status, aquaculture model, cattle type, cattle age, longitude, and latitude) were explored to identify covariates of virome and bacterial community variation, respectively, by calculating the association between continuous or categorical phenotypes and virus and bacteria abundance with multiple linear regression analysis and binary logistic regression analysis in IBM SPSS Statistics, version 22.0 (IBM SPSS Inc., USA). Various environment and host conditions included categorical variables: clinical status (diarrhea, health), aquaculture model (intensive, non-intensive), cattle type (dairy cow, beef cattle), breed (Angus, Holstein, Simmental), and continuous variables: cattle age (6–1,003 days) and geography location (eastern Heilongjiang: Hegang [latitude: 47.33°N; longitude: 130.27°E], Jiamusi [latitude: 46.80°N; longitude: 130.36°E], Shuangyashan [latitude: 46.64°N; longitude: 131.15°E], Jixi [latitude: 45.30°N; longitude: 130.97°E]; western Heilongjiang: Qiqihar [latitude: 47.34°N; longitude: 123.95°E], Suihua [latitude: 46.63°N; longitude: 126.99°E], Daqing [latitude: 46.59°N; longitude: 125.11°E]; southern Heilongjiang: Harbin [latitude: 45.75°N; longitude: 126.64°E], Mudanjiang [latitude: 44.58°N; longitude: 129.61°E]; northern Heilongjiang: Daxinganling [latitude: 52.33°N; longitude: 124.71°E], Heihe [latitude: 50.24°N; longitude: 127.49°E], Yichun [latitude: 47.72°N; longitude: 128.89°E]). The significance level in all analyses was 5%, with a confidence interval of 95%. The correlations of microorganisms (viruses or bacteria) with clinical status, aquaculture model, and cattle type were detected with 2 × 2 contingency tables and χ2 test. In order to measure the correlation between microbiome composition among different hosts and environmental factors, we performed virus-to-virus correlation or bacterial-to-bacterial correlation identification. The plot was generated using R software (v4.2.2) package “corrplot” (v0.92) and “ggplot2” (v3.4.2) through Hiplot Pro (https://hiplot.com.cn/). Descriptive statistics were calculated for virus or bacteria variables with various factors evaluated. Categorical data were analyzed with a χ2 test. Differences in values were considered statistically significant (*P* < 0.05) or highly significant (*P* < 0.01) and were included in chord diagram analysis. Correlations between gut virus and bacteria variation were calculated through the Spearman correlation test. Correlation coefficient was calculated, while statistical significance was determined for all pairwise comparisons. Only statistically significant correlations with correlation coefficients >0.26 were plotted. The correlation network was visualized via Cytoscape.

### PCR confirmation and analysis of the 10 bovine enteric viruses

PCR confirmation was performed for the BKV, BoNoV, BoNeV, BoPV, BRV, BCoV, BToV, BoAstV, BEV, and BVDV in the 1,120 fecal samples in our study. The primer sequences are listed in Table 3. RT-PCR conditions were the same as described in the corresponding references. The detection results of BCoV had been reported in a previous study (66). The positive rate of 10 viruses in Heilongjiang Province was carried out using GraphPad Prism 8.00 (GraphPad Software, Inc., USA). The map of Heilongjiang Province was drawn with an online map-generation website (http://pixelmap.amcharts.com/) (67). Co-infections of identified viruses were performed using the online software Hiplot (https://hiplot-academic.com/) (68). The different factors were explored to identify covariates of virome variation, respectively, by calculating the association between continuous or categorical phenotypes and 10 bovine enteric viruses with binary logistic regression analysis in IBM SPSS Statistics, version 22.0 (IBM SPSS Inc., USA). The positive rate between different factors for 10 bovine enteric viruses was performed by GraphPad Prism 8.00.

**TABLE 3:** The primers used in this study

Primer name	Primer sequence (5′ → 3′)	Primer reference
BKV-F	TGGATTACAAGAATGTTTTGATGC	(69)
BKV-R	TGTTGTTATGATGGTGTTGA	
BToV-F	GAGAAAGAGCCAAGATGAATT	(70)
BToV-R	CTTACATGGAGACACTCAACCA	
BoNoV-F	GTCGACGGYCTKGTSTTCCT	(47)
BoNoV-R	CACAGCGACAAATCATGAAA	
BoNeV-F	CAGCCCGTCTGGGTGAAT	(71)
BoNeV-R	CTGGATTGTTCTGACTTCGG	
BoPV-F	CTTTTTCCCCCTCTTGTAAC	(72)
BoPV-R	TTAGCCGCATTCAGGGGCCTGGAG	
BoAstV-F	GCACGTTCGTCCTCGATGT	(73)
BoAstV-R	ATACGTTTGGCCTCGCTCACA	
BoAstV-nF	CCGTCAGATAATATGCCCCGAT	
BoAstV-nR	GTCCTCCCCTCCAAAGACGAT	
BRV-F	TTGATGGGTACGATGTGGCT	(74)
BRV-R	CTGGTGTCATATTTGGTGGTCT	
BVDV-F	GGTAGTCGTCAGTCAGTGGTTCGAC	(75)
BVDV-R	CATGTGCCATGTACAGCAGAGAT	
BEV-F	ACCTTTGTACGCCTGTTTTCC	(76)
BEV-R	GATTAGCAGCATTCACGGC	

### Sequence alignment and phylogenetic analyses

The nucleotide (nt) and deduced amino acid (aa) sequences were analyzed using the MegAlign software (DNASTAR, Madison, WI, USA) (77). The alignment results were analyzed by using the MegAlign tool in the Lasergene DNASTAR version 5.06 software (DNASTAR Inc., USA). The nt sequences of the genomes and the deduced aa sequences of the ORFs were compared to those of representative members of related viral species or genera using ClustalW by multiple sequence alignment. Phylogenetic trees were constructed using the maximum likelihood (ML) method and a bootstrap value of 1,000 by MEGA 6 program. The alignment sequences were constructed as Bayes evolutionary trees using MrBayes 3.2.7 program with set cutoff frequency (default value 0.10). We used the “sump” and “sumt” commands to get more detailed diagnostic information after the run has completed. The phylogenetic tree was annotated with the Interactive Tree Of Life (iTOL) software (http://itol.embl.de/), an online tool for the display and annotation of phylogenetic trees (78).

### Statistical analysis

Statistical analysis was performed in R (version 3.5.1). Unless otherwise stated, analysis was performed using a Wilcoxon test to compare two groups and Kruskal-Wallis for more than two groups. Statistical significance is represented by *P value < 0.05, **P value < 0.01, ***P value < 0.001, and ****P value < 0.0001.

## Data Availability

The raw sequence data reported in this paper have been deposited in the Genome Sequence Archive in National Genomics Data Center, China National Center for Bioinformation/Beijing Institute of Genomics, Chinese Academy of Sciences (GSA: CRA011306) that are publicly accessible at https://ngdc.cncb.ac.cn/gsa (79, 80). Relevant pathogen genome/gene sequences have been deposited in GenBank (see Table S3 for accession numbers).

## SUPPLEMENTAL MATERIAL

Figure S1 msystems.00842-23-s0001.tif Sequence analysis and genetic evolution of identified BKV.

Supplemental Information msystems.00842-23-s0002.docx Captions for supplemental material.

Table S1 msystems.00842-23-s0003.xlsx Details of all samples in this study.

Table S2 msystems.00842-23-s0004.xlsx Viral library information of cattle fecal samples in this study.

Table S3 msystems.00842-23-s0005.docx GenBank accession numbers of novel viral genomes identified in this study.

Table S4 msystems.00842-23-s0006.xlsx Bacterial library information of cattle fecal samples in this study.

Table S5 msystems.00842-23-s0007.docx Correlation analysis between different factors and virome at the family level and the bacterial community at the genus level.

Table S6 msystems.00842-23-s0008.docx The positive rate of 10 bovine enteric viruses identified in this study in different cities (regions).

Table S7 msystems.00842-23-s0009.xlsx The positive distribution information of 10 bovine enteric viruses of all samples.

Table S8 msystems.00842-23-s0010.docx Nucleotide and deduced amino acid homology of the BKV5/2021/CHN strain in this study and other representative kobuviruses in GenBank.

ASM does not own the copyrights to Supplemental Material that may be linked to, or accessed through, an article. The authors have granted ASM a non-exclusive, world-wide license to publish the Supplemental Material files. Please contact the corresponding author directly for reuse.
