# Supplementary figures and images for: A survey of fecal virome and bacterial community of the diarrhea-affected cattle in northeast China reveals novel disease-associated ecological risk factors
Source: mSystems. 2023 Dec 18;9(1):e00842-23. doi: 10.1128/msystems.00842-23 (PMC10804951; doi:10.1128/msystems.00842-23)

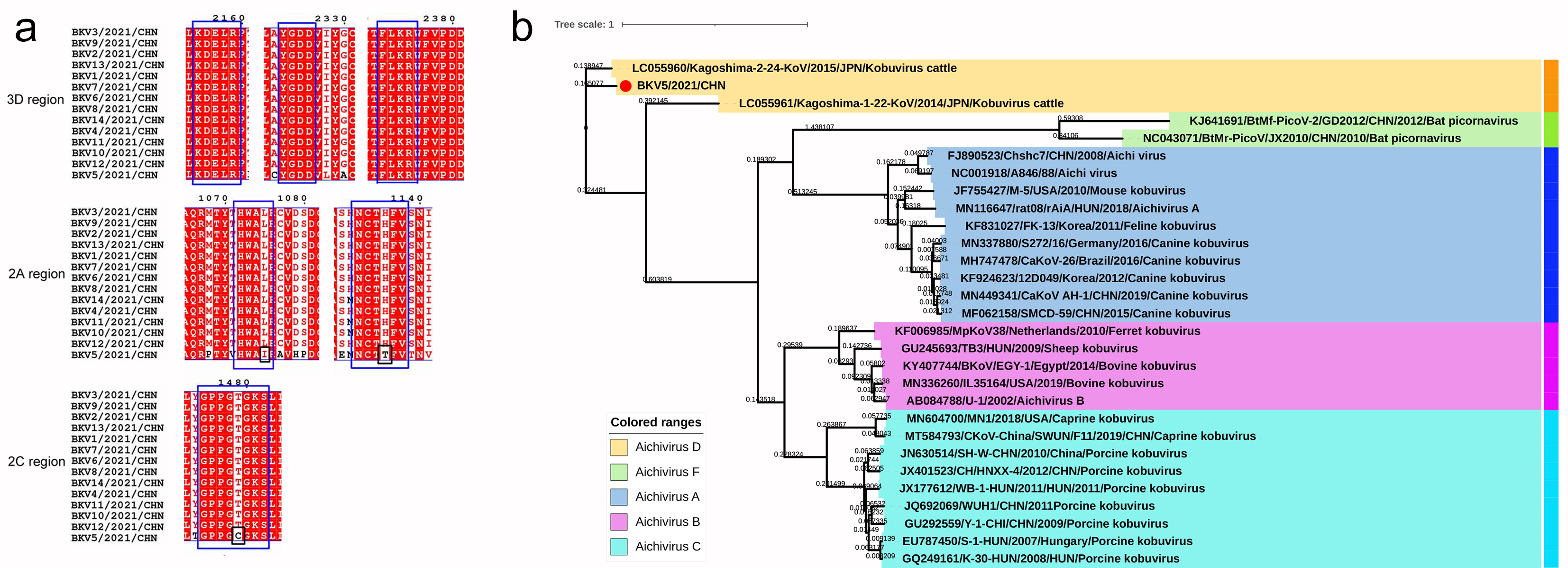

Supplement: Figure S1 — Sequence analysis and genetic evolution of identified BKV. [file msystems.00842-23-s0001.tif]
