# Supplementary material for: A survey of fecal virome and bacterial community of the diarrhea-affected cattle in northeast China reveals novel disease-associated ecological risk factors
Source: mSystems. 2023 Dec 18;9(1):e00842-23. doi: 10.1128/msystems.00842-23 (PMC10804951; doi:10.1128/msystems.00842-23)
Supplement: Supplemental Information — Captions for supplemental material. [file msystems.00842-23-s0002.docx]

**Supplementary Information**

**Table S1.** Details of all samples in this study.

**Table S2.** Viral library information of cattle fecal samples in this study.

**Table S3.** GenBank accession numbers of novel viral genomes identified in this study.

**Table S4.** Bacterial library information of cattle fecal samples in this study.

**Table S5.** Correlation analysis between different factors and virome at the family level and the bacterial community at the genus level.

**Table S6.** The positive rate of 10 bovine enteric viruses identified in this study in different cities (regions).

**Table S7.** The positive distribution information of 10 bovine enteric viruses of all samples.

**Table S8.** Nucleotide and deduced amino acid homology of the BKV5/2021/CHN strain in this study and other representative kobuviruses in GenBank.

**FIGURE LEGENDS**

**Figure S1.** Sequence analysis and genetic evolution of identified BKV. (**a)** The amino acid sequence analysis of identified BKV. (**b)** Bayesian phylogenetic tree of BKV VP1 sequences.
