# Supplementary material for: A survey of fecal virome and bacterial community of the diarrhea-affected cattle in northeast China reveals novel disease-associated ecological risk factors
Source: mSystems. 2023 Dec 18;9(1):e00842-23. doi: 10.1128/msystems.00842-23 (PMC10804951; doi:10.1128/msystems.00842-23)
Supplement: Table S3 — GenBank accession numbers of novel viral genomes identified in this study. [file msystems.00842-23-s0005.docx]

**Table** **S3. GenBank accession numbers of novel viral genomes identified in this study.**

BoNeV1/2021/CHN: ON133004

BoNeV2/2021/CHN: ON133005

BoNeV3/2021/CHN: ON133006

BoNeV4/2021/CHN: ON133007

BoNeV5/2021/CHN: ON787767

BoNeV6/2021/CHN: ON133008

BoNeV7/2021/CHN: ON133009

BoNeV8/2021/CHN: ON133010

BoNeV9/2021/CHN: ON133011

BoNeV10/2021/CHN: ON787768

BoNeV11/2021/CHN: OP272996

BoNeV12/2021/CHN: OP234432

BoNeV13/2021/CHN: OP272997

BoNeV14/2021/CHN: OP272998

BoNeV15/2021/CHN: OP272999

BoNeV16/2021/CHN: OP272930

BoAstV1/2021/CHN: ON624251

BoAstV2/2021/CHN: ON624252

BoAstV3/2021/CHN: ON624253

BoAstV4/2021/CHN: ON624254

BoAstV5/2021/CHN: ON624255

BoAstV6/2021/CHN: ON624256

BoAstV7/2021/CHN: ON624257

BoAstV8/2021/CHN: ON624258

BoAstV9/2021/CHN: ON624259

BoAstV10/2021/CHN: ON624260

BoAstV11/2021/CHN: ON624261

BoAstV12/2021/CHN: ON624262

BoAstV13/2021/CHN: ON624263

BoAstV14/2021/CHN: ON624264

BoAstV15/2021/CHN: ON624265

BoAstV16/2021/CHN: ON624266

BoAstV17/2021/CHN: ON624267

BoAstV18/2021/CHN: ON624268

BoAstV19/2021/CHN: ON624269

BoAstV20/2021/CHN: ON624270

BoAstV21/2021/CHN: ON624271

BoAstV22/2021/CHN: ON624272

BoAstV23/2021/CHN: ON624273

BoAstV24/2021/CHN: ON624274

BoAstV25/2021/CHN: ON624275

BoAstV26/2021/CHN: ON624276

BoAstV27/2021/CHN: ON624277

BoAstV28/2021/CHN: ON624278

BoAstV29/2021/CHN: ON624279

BoAstV30/2021/CHN: ON624280

BoAstV31/2021/CHN: ON624281

BoAstV32/2021/CHN: ON624282

BoAstV33/2021/CHN: ON624283

BoAstV34/2021/CHN: ON624284

BoAstV35/2021/CHN: OP331312

BoAstV36/2021/CHN: ON682271

BoAstV37/2021/CHN: ON682272

BoAstV38/2021/CHN: ON682273

BoAstV39/2021/CHN: ON682274

BoAstV40/2021/CHN: ON682275

BoAstV41/2021/CHN: ON682276

BoAstV42/2021/CHN: ON682277

BoAstV43/2021/CHN: ON682278

BoAstV44/2021/CHN: ON682279

BoAstV45/2021/CHN: ON682280

BoAstV46/2021/CHN: ON682281

BoAstV47/2021/CHN: ON682282

BoAstV48/2021/CHN: ON682283

BoAstV49/2021/CHN: ON682284

BoAstV50/2021/CHN: ON682285

BoAstV51/2021/CHN: ON682286

BoAstV52/2021/CHN: ON682287

BoAstV53/2021/CHN: ON682288

BoAstV54/2021/CHN: ON682289

BoAstV55/2021/CHN: ON6822290

BoAstV56/2021/CHN: ON682291

BoAstV57/2021/CHN: ON682292

BoAstV58/2021/CHN: ON682293

BoAstV59/2021/CHN: ON682294

BoAstV60/2021/CHN: ON682295

BoAstV61/2021/CHN: ON682296

BoAstV62/2021/CHN: ON682297

BoAstV63/2021/CHN: ON682298

BoAstV64/2021/CHN: ON682299

BoAstV65/2021/CHN: ON682300

BoAstV66/2021/CHN: ON682301

BoAstV67/2021/CHN: ON682302

BoAstV68/2021/CHN: ON682303

BoAstV69/2021/CHN: ON885949

BoAstV70/2021/CHN: ON885950

BoAstV71/2021/CHN: ON885951

BoAstV72/2021/CHN: ON885952

BKV1/2021/CHN: ON075050

BKV2/2021/CHN: ON075051

BKV3/2021/CHN: ON075052

BKV4/2021/CHN: ON075053

BKV5/2021/CHN: ON730709

BKV6/2021/CHN: ON075054

BKV7/2021/CHN: ON075055

BKV8/2021/CHN: ON075056

BKV9/2021/CHN: ON168737

BKV10/2021/CHN: ON168738

BKV11/2021/CHN: ON168739

BKV12/2021/CHN: ON168740

BKV13/2021/CHN: ON168741

BKV14/2021/CHN: ON168742

BVDV1/2021/CHN: ON997623

BEV1/2021/CHN: ON997621

BEV2/2021/CHN: ON997622

BEV3/2021/CHN: ON986116

BEV4/2021/CHN: ON986117

BEV5/2021/CHN: ON986118

BEV6/2021/CHN: ON986119

BEV7/2021/CHN: ON986120

BEV8/2021/CHN: ON624115

BEV9/2021/CHN: ON986121

BEV10/2021/CHN: ON986122

BoPV1/2021/CHN: ON148337

BoPV2/2021/CHN: ON148338

BoPV3/2021/CHN: ON148339

BoPV4/2021/CHN: ON148340

BoPV5/2021/CHN: ON168930

BoPV6/2021/CHN: ON168931

BoPV7/2021/CHN: ON168932

BoPV8/2021/CHN: ON168933

BoPV9/2021/CHN: OP062271

BoPV10/2021/CHN: OP221745

BoPV11/2021/CHN: OP235943

BoPV12/2021/CHN: OP235944

BoPV13/2021/CHN: OP235945

BoPV14/2021/CHN: OP263973

BoPV15/2021/CHN: OP263974

BoPV16/2021/CHN: OP263975

BoPV17/2021/CHN: OP263976

BoPV18/2021/CHN: OP263977

BoPV19/2021/CHN: OP263978

BoPV20/2021/CHN: OP263979

BoPV21/2021/CHN: OP263980

BoPV22/2021/CHN: OP263981

BoPV23/2021/CHN: OP263982

BoPV24/2021/CHN: OP263983

BoPV25/2021/CHN: OP263984

BoPV26/2021/CHN: OP302805

BoPV27/2021/CHN: OP302806

BCoV1/2021/CHN: ON142315

BCoV2/2021/CHN: ON093194

BCoV3/2021/CHN: ON142316

BCoV4/2021/CHN: ON142317

BCoV5/2021/CHN: ON142318

BCoV6/2021/CHN: ON142319

BToV1/2021/CHN: ON142321

BToV2/2021/CHN: ON142322

BToV3/2021/CHN: ON165540

BToV4/2021/CHN: ON165541

BToV5/2021/CHN: ON165542

BToV6/2021/CHN: ON165543

BRV1/2021/CHN

VP4: OP169136

VP7: OP169140

BRV2/2021/CHN

VP4: OP169136

VP7: OP169141

BRV3/2021/CHN

VP4: OP169137

VP7: OP169142

BRV4/2021/CHN

VP4: OP169138

VP7: OP169143

BRV5/2021/CHN

VP4: OP169139

VP7: OP169144

Dicistroviridae

H-A/14: OP186470

I-JX-MX/2: OP186471

I-QQ-GM/12: OP186472

I-SH-YZY/6: OP186473

R-HH-LM/12: OP287010

R-MDJ-HS-2/6: OP287011

R-QQ-FQ/18: OP287012

R-SYS-XD/9: OP287013

R-YC-YM/18: OP287014

Picornaviridae

I-HH-HZY/39: OP245250

I-QQ-QM/20: OP245251

I-HG-JC/61: OP245252

I-HH-XA/9: OP245253

I-HRB-FY/57: OP245254

I-QQ-XH/21: OP245255

R-MDJ-HS-1/30: OP245256

R-QQ-FQ/28: OP245257

Genomoviridae sp.

R-DXAL-HM-2/107: OQ779166

R-DXAL-HM-2/110: OQ779167

R-DXAL-HM-2/112: OQ779168

R-DXAL-SK-1/263: OQ779169

R-DXAL-SK-2/48: OQ779170

R-DXAL-HM-3/128: OQ779171

R-DXAL-HM-3/131: OQ779172

R-DXAL-TH/163: OQ779173

R-HG-BQL-1/124: OQ779174

R-MDJ-HS-1/119: OQ779175

R-MDJ-HS-3/191: OQ779176

R-QQ-FQ/84: OQ779177

R-SYS-XD/55: OQ779178

R-YC-YM/109: OQ779179

R-YC-YM/121: OQ779180

H-R/122: OQ792003

I-HH-DS/115: OQ792004

I-HH-DS/117: OQ792005

I-HH-DS/118: OQ792006

I-HH-FY/105: OQ792007

I-HH-MX/101: OQ792008

I-HH-MX/107: OQ792009

I-JX-XX/274: OQ792010

I-JX-YQ/140: OQ792011

I-QQ-HD/270: OQ792012

I-QQ-QM/104: OQ792013

I-QQ-XH/68: OQ792014

R-DXAL-HM-1/65: OQ792015

R-DXAL-HM-1/71: OQ792016

R-DXAL-HM-1/72: OQ792017

H-R/99: OQ791987

I-DQ-YX/64: OQ791988

I-HH-JS/142: OQ791989

I-HH-JS/161: OQ791990

I-HH-MX/77: OQ791991

I-HRB-FY/258: OQ791992

I-JX-XX/294: OQ791993

I-JX-YQ/132: OQ791994

I-QQ-GM/58: OQ791995

I-QQ-HD/280: OQ791996

R-MDJ-HS-1/128: OQ791997

H-A/98: OQ791998

H-A/103: OQ791999

H-B-AGS/82: OQ792000

H-B-AGS/115: OQ792001

H-B-AGS/116: OQ792002

H-B-C/50: OQ835317

H-B-C/54: OQ835318

H-B-C/55: OQ835319

H-B-XMTR/109: OQ835320

H-B-XMTR/116: OQ835321

H-B-XMTR/119: OQ835322

H-B-XMTR/120: OQ835323

H-C-H/204: OQ835324

H-C-HST/144: OQ835325

H-I/136: OQ835326

H-I/137: OQ835327

H-I/140: OQ835328

H-I/144: OQ835329

H-I/116: OQ835330

H-R/117: OQ835331

H-R/121: OQ835332
