# Supplementary material for: A survey of fecal virome and bacterial community of the diarrhea-affected cattle in northeast China reveals novel disease-associated ecological risk factors
Source: mSystems. 2023 Dec 18;9(1):e00842-23. doi: 10.1128/msystems.00842-23 (PMC10804951; doi:10.1128/msystems.00842-23)
Supplement: Table S5 — Correlation analysis between different factors and virome at the family level and the bacterial community at the genus level. [file msystems.00842-23-s0007.docx]

**Table** **S5. Correlation analysis between different infection factors and the virome at the family level and the bacterial community at the genus level.**

| Virues | Clinical status (diarrhea) | Aquaculture model (D-intensive) | Cattle type (D-dairy cow) |
| --- | --- | --- | --- |
| Astroviridae | 0.959 | **0.005** | 0.199 |
| Caliciviridae | 0.099 | 0.134 | **0.03** |
| Coronaviridae | **0.035** | 0.793 | 0.325 |
| Genomoviridae | 0.132 | 0.612 | **0.049** |
| Herpesviridae | 0.944 | 0.676 | **0.006** |
| Picornaviridae | **0.001** | **0.044** | 0.438 |
| Reoviridae | 0.351 | 0.121 | **0.014** |
| Siphoviridae | 0.064 | 0.075 | **0.022** |
| Totiviridae | 0.955 | 0.135 | **0.022** |
| Virgaviridae | 0.862 | **0.018** | **0** |
| Collinsella | 0.876 | **0.006** | **0** |
| Planomicrobium | 1 | **0.004** | **0** |
| Trichococcus | 0.233 | **0.034** | **0.001** |
| Carnobacterium | 1 | **0.004** | **0.001** |
| Subdoligranulum | 1 | **0.003** | **0.002** |
| Chryseobacterium | 1 | **0.013** | **0.002** |
| Butyricicoccus | 1 | **0** | **0** |
| Olsenella | 1 | **0.005** | **0.001** |
| Aerococcus | 0.579 | 0.196 | **0.037** |
| Staphylococcaceae_Staphylococcus | 0.057 | **0.037** | **0.002** |
| Erysipelotrichaceae_Clostridium | 1 | **0.037** | **0.01** |
| Lactococcus | 0.218 | **0.022** | **0.001** |
| Faecalibacterium | 0.318 | **0.001** | **0.001** |
| Actinomyces | 0.052 | **0.001** | **0** |
| Acidovorax | 0.17 | 0.237 | **0.048** |
| Mogibacterium | 0.663 | 0.129 | **0.039** |
| SMB53 | 0.089 | 0.088 | **0.02** |

**Note.** Differences in values were considered statistically significant or highly significant at *p* < 0.05 or *p* < 0.01, respectively. statistically significant or highly significant in bold. D-intensive is diarrhea cattle with intensive farming, D-dairy cow is diarrhea dairy cow.
