# Supplementary material for: A survey of fecal virome and bacterial community of the diarrhea-affected cattle in northeast China reveals novel disease-associated ecological risk factors
Source: mSystems. 2023 Dec 18;9(1):e00842-23. doi: 10.1128/msystems.00842-23 (PMC10804951; doi:10.1128/msystems.00842-23)
Supplement: Table S6 — The positive rate of 10 bovine enteric viruses identified in this study in different cities (regions). [file msystems.00842-23-s0008.docx]

**Table** **S6. The positive rate of 10 bovine enteric viruses identified in this study in different cities (regions).**

| **City (region)** | **BKV** | **BToV** | **BoNoV** | **BoNeV** | **BoPV** | **BRV** | **BoAstV** | **BCoV** | **BEV** | **BVDV** |
| --- | --- | --- | --- | --- | --- | --- | --- | --- | --- | --- |
|  | Pos/Tot (%) | Pos/Tot (%) | Pos/Tot (%) | Pos/Tot (%) | Pos/Tot (%) | Pos/Tot (%) | Pos/Tot (%) | Pos/Tot (%) | Pos/Tot (%) | Pos/Tot (%) |
| **Jixi** | 17/119 (14.29%) | 7/119 (5.88%) | 2/119 (1.68%) | 7/119 (5.88%) | 12/119 (10.08%) | 16/119 (13.45%) | 24/119 (20.17%) | 10/119 (8.40%) | 1/119 (0.84%) | 00/119 (0.00%) |
| **Heihe** | 44/240 (18.33%) | 11/240 (4.58%) | 3/240 (1.25%) | 30/240 (12.50%) | 12/240 (5.00%) | 14/240 (5.83%) | 24/240 (10.00%) | 34/240 (14.17%) | 2/240 (0.83%) | 10/240 (4.17%) |
| **Qiqihar** | 6/79 (7.59%) | 6/79 (7.59%) | 5/79 (6.32%) | 1/79 (1.27%) | 7/79 (8.86%) | 12/79 (15.19%) | 4/79 (5.06%) | 51/79 (64.56%) | 0/79 (0.00%) | 1/79 (1.27%) |
| **Daqing** | 5/69 (7.25%) | 2/69 (2.90%) | 0/69 (0.00%) | 2/69 (2.90%) | 0/69 (0.00%) | 1/69 (1.45%) | 8/69 (11.59%) | 9/69 (13.04%) | 0/69 (0.00%) | 0/69 (0.00%) |
| **Yichun** | 17/72 (23.61%) | 0/72 (0.00%) | 5/72 (6.94%) | 9/72 (12.50%) | 3/72 (4.17%) | 6/72 (8.33%) | 6/72 (8.33%) | 1/72 (1.39%) | 0/72 (0.00%) | 11/72 (15.28%) |
| **Suihua** | 1/45 (2.22%) | 2/45 (4.44%) | 9/45 (20.00%) | 1/45 (2.22%) | 2/45 (4.44%) | 2/45 (4.44%) | 2/45 (4.44%) | 0/45 (0.00%) | 0/45 (0.00%) | 6/45 (13.33%) |
| **Harbin** | 7/97 (7.22%) | 5/97 (5.15%) | 6/97 (6.19%) | 5/97 (5.15%) | 9/97 (9.28%) | 10/97 (10.31%) | 9/97 (9.28%) | 3/97 (3.09%) | 0/97 (0.00%) | 2/97 (0.02%) |
| **Hegang** | 9/142 (6.34%) | 7/142 (4.93%) | 5/142 (3.52%) | 9/142 (6.34%) | 2/142 (1.41%) | 8/142 (5.63%) | 12/142 (8.45%) | 9/142 (6.34%) | 6/142 (0.04%) | 0/142 (0.00%) |
| **Jiamusi** | 0/4 (0.00%) | 0/4 (0.00%) | 0/4 (0.00%) | 0/4 (0.00%) | 0/4 (0.00%) | 1/4 (25%) | 0/4 (0.00%) | 0/4 (0.00%) | 1/4 (25.00%) | 0/4 (0.00%) |
| **Shuangyashan** | 7/49 (14.29%) | 3/49 (6.12%) | 8/49 (16.33%) | 3/49 (6.12%) | 2/49 (4.08%) | 10/49 (20.41%) | 7/49 (14.29%) | 2/49 (4.08%) | 2/49 (4.08%) | 0/49 (0.00%) |
| **Daxinganling** | 19/137 (13.87%) | 4/137 (2.92%) | 8/137 (5.84%) | 1/137 (0.73%) | 2/137 (1.46%) | 8/137 (5.83%) | 13/137 (9.49%) | 2/137 (1.46%) | 6/137 (4.38%) | 2/137 (1.46%) |
| **Mudanjiang** | 3/67 (4.48%) | 5/67 (7.46%) | 2/67 (2.99%) | 8/67 (11.94%) | 0/67 (0.00%) | 3/67 (4.48%) | 5/67 (7.46%) | 3/67 (4.48%) | 0/67 (0.00%) | 0/67 (0.00%) |
| **Total** | 135/1120 (12.05%) | 52/1120 (4.64%) | 53/1120 (4.73%) | 76/1120 (6.79%) | 52/1120 (4.64%) | 91/1120 (8.13%) | 114/1120 (10.18%) | 124/1120 (11.07%) | 18/1120 (1.60%) | 32/1120 (2.86%) |
