# Supplementary material for: A survey of fecal virome and bacterial community of the diarrhea-affected cattle in northeast China reveals novel disease-associated ecological risk factors
Source: mSystems. 2023 Dec 18;9(1):e00842-23. doi: 10.1128/msystems.00842-23 (PMC10804951; doi:10.1128/msystems.00842-23)
Supplement: Table S8 — Nucleotide and deduced amino acid homology of the BKV5/2021/CHN strain in this study and other representative kobuviruses in GenBank. [file msystems.00842-23-s0010.docx]

**Table S8. Nucleotide and deduced amino acid homology of the BKV5/2021/CHN strain in this study and other representative kobuviruses in GenBank.**

| Strain | Host | GenBank  accession number | Genome  nucleotide identities | Amino acid identities | | | |
| --- | --- | --- | --- | --- | --- | --- | --- |
|  |  |  |  | Complete polyprotein | P1 | 2C | 3CD |
| AN211D/A | Dog | JN387133 | 57.1% | 49.3% | 45.0% | 52.4% | 60.6% |
| U-1/B | Bovine | AB084788 | 57.8% | 52.0% | 52.9% | 54.1% | 61.3% |
| F11/C | Caprine | MT584793 | 57.7% | 51.5% | 51.4% | 56.5% | 59.8% |
| S-1-HUN/C | Porcine | EU787450 | 56.0% | 51.1% | 52.7% | 53.5% | 59.7% |
| 2-24-kov/D | Cattle | LC055960 | 81.1% | 85.7% | 69.0% | 97.9% | 96.1% |
| 1-22-kov/D | Cattle | LC055961 | 66.7% | 65.4% | 71.7% | 61.6% | 72.3% |
| BtMf-picov-2/F | Bat | KJ641691 | 50.7% | 43.7% | 43.7% | 43.6% | 50.5% |
